# Providing maceration protocols for xylem and phloem research

**DOI:** 10.3389/fpls.2025.1740174

**Published:** 2026-01-22

**Authors:** Eunice Romero, Mark Olson, Alí Segovia-Rivas, Jarmila Pittermann, Radek Jupa

**Affiliations:** 1 Department of Physical Geography and Geoecology, Faculty of Science, Charles University, Prague, Czechia; 2 Biology Institute, Botany Department, National Autonomous University of Mexico, Mexico City, Mexico; 3 Department of Ecology and Evolutionary Biology, University of California, Santa Cruz, CA, United States; 4 Department of Experimental Biology, Masaryk University, Brno, Czechia

**Keywords:** allometry, conduit diameter, dissociates, Jeffrey, quantitative wood anatomy, sieve tube elements, tracheid and vessel length

## Abstract

Maceration technique allows isolating and studying the structure of plant cells, providing essential insights into plant physiology and responses to environmental factors. Despite its importance, the methods sections of publications often lack sufficient detail to properly apply maceration technique, preventing broader applications beyond industrial and wood identification studies. Here we describe maceration protocols (P), as guidelines, to allow successful cell separation, in woody and herbaceous plants, for further studying the structure of vascular tissues (xylem and phloem), using Franklin’s solution instead of the traditional, more toxic Jeffrey’s. We included sample preparation, equipment and chemicals, recommended stains and means of slide preparation that ensure clear observation and imaging, and identified potential pitfalls and safety practices. P1 provides a simple xylem and phloem maceration technique, with non-permanent slides preparation, suitable for observation under light, fluorescence or confocal microscopy. P2 and P3 are suitable for producing permanent slides of macerated xylem cells. P2 has been successfully used for macerating xylem of angiosperms and gymnosperms from at least 34 families and 25 orders, with densities ranging widely. P3 uses minimal substances and time for dehydration and staining. These flexible protocols may contribute to unlocking the potential of maceration technique to advance research in ecology and evolution.

## 1 Introduction

Xylem and phloem represent two vascular tissues of plant vital functional importance. Xylem facilitates long-distance transport of water from roots to leaves and also serves as an important mechanical support of the plant body, while phloem primarily distributes carbohydrates and other compounds across the plant body. The study of structural properties of xylem and phloem provides key insights into the evolution of vascular plants (Sperry, 2003; Pittermann, 2010) and is crucial for plant responses to the environmental cues and species ecological distribution in diverse biomes (Hacke and Sperry, 2001; Choat et al., 2018; Salmon et al., 2019; Wang et al., 2025; Hacke, 2015; Schulz, 1998). Moreover, wood serves as a key resource for industries such as paper manufacturing, making the study of its cellular structure essential (Helmling et al., 2016; Li et al., 2023).

Anatomical research on xylem and phloem is frequently challenging due to their notably heterogeneous cellular composition. Both tissues are composed of multiple cell types that largely differ in their size, cell wall thickness and composition. Moreover, the structure of xylem and phloem notably differs between gymnosperm and angiosperm species. In gymnosperms, xylem is dominated by typically long, narrow and thick-walled imperforate tracheary elements (ITEs) that serve both conductive and supportive functions (Schulz, 1998; Hacke 2015). Angiosperms, by contrast, possess vessels in addition to ITEs. Vessels are formed from vessel elements, which are shorter and wider than ITEs, aligned end-to-end along the longitudinal axis, and separated by perforation plates. These vessel elements are interconnected by bordered pits, enabling efficient water transport while also restricting the spread of embolisms.

Phloem conduits in both groups share certain morphological parallels with xylem conduits (Knoblauch and Oparka, 2012; Hardtke, 2023). Gymnosperm phloem is composed primarily of sieve cells that facilitate transport processes. Sieve cells are often associated with albuminous cells. In angiosperms, phloem contains sieve tubes formed by sieve tube elements, which are closely associated with companion cells. Companion cells, similar to albuminous cells in gymnosperms, provide essential metabolic support to sieve tube cells. Unlike xylem pits, phloem conduits are interconnected by sieve areas containing numerous pores. In angiosperms, these pores are aggregated into sieve plates located at the ends of sieve tube elements, allowing for highly efficient translocation of photosynthates. In contrast to xylem conduits, which are composed of thick secondary cell walls, phloem conduits in both gymnosperms and angiosperms have substantially thinner, mostly primary cell walls. Beyond conduits, both xylem and phloem in angiosperms typically contain thick-walled fibers that provide mechanical strength and axial parenchyma cells that contribute to storage and metabolic activity. Radial parenchyma cells form rays that span across xylem and phloem, creating pathways for lateral transport and functional integration between the two tissues (Carlquist, 2018). The anatomical differences between the xylem and phloem of gymnosperm and angiosperm species may necessitate specific approaches to accurately study the structure of both tissues across a wide spectrum of species.

Cross-sectioning followed by microscopic observations are one of the most common methods employed to study xylem and phloem structure (Carlquist, 1975; Pace, 2019; Fonti et al., 2025). However, this widely used approach has limitations in identification of individual cell types and assessing their allometry. For example, imperforate tracheary elements in xylem or sieve cells in phloem often vary in diameter along their length, tapering from a wide central region toward narrower tips. Because their diameters are conventionally measured at the point of maximum width, it is not possible to determine this from a cross-section, as there is no way of knowing whether the section intersects the widest point, or lies above or below it. In addition, when the diameters of conductive imperforate tracheary elements overlap with those of vessel elements, the two cell types cannot be distinguished in cross-section. As a result, measurements can be significantly biased because either a large number of imperforate tracheary elements will be included in the sample, dragging the distribution down erroneously, or a large number of narrow vessels will be excluded, which artificially skews the distribution upward. These limitations inherent to cross-sectioning may frequently lead to misidentifications of wood cell types and bias results of quantitative wood anatomy, particularly when cell type assignment is based solely on the cell diameter (Wegner et al., 2013; but see Katzenmaier et al., 2023).

Radial and tangential sections provide additional information about cell morphology along with cross-sections, and are useful for accurate identification of individual cell types. Longitudinal sections are also essential for measuring many of the structural traits, such as cell pitting, ray density (Arenas-Navarro et al., 2023; Romero et al., 2020), numbers of cells in parenchyma strands, and are more suitable for observation of fine structures like perforation plates of xylem vessel members, or sieve plates of phloem sieve tube members (Wheeler and Baas, 1998; Liesche et al., 2017). Yet, longitudinal sections may not allow quantitative analyses of the ultrastructure of perforation or sieve plates since most of the plates may not be complete due to sample sectioning. Also, measuring the length of imperforate tracheary elements or sieve cells in radial sections is complicated and imprecise, because even small longitudinal undulations, including those due to intrusive growth along the axis, can move the tips of the long cells out of the plane of sectioning. Even if cells were perfectly aligned axially, small misalignments of the plane of sectioning can still make the tips of individual cells difficult to locate.

Consequently, in addition to the three standard sections, macerations are essential for accurate quantitative characterization of cells forming xylem and phloem tissues. In industrial contexts, maceration has been applied to enhance the prediction of growth and yield in pulp and paper production (Dahlen et al., 2021), and to assess the physical and mechanical properties of hardwood species for wood production (Kiaei and Samariha, 2011). Macerations are also useful for studying biomechanics through measuring fiber lengths (Oladi et al., 2025). Beyond its industrial, forestry and forest economic applications, macerations have been widely used for xylem identification and anatomical descriptions (IAWA Committee, 1989).

However, although macerations are promising to be a powerful tool in ecological and evolutionary research, their potential in these areas is still largely unfulfilled. In functional ecology understanding cell size, arrangement and pit properties helps to explain xylem transport properties. Maceration can be especially useful for measuring lengths of vessel elements and tracheids needed to estimate xylem transport efficiency (Pittermann et al., 2005; Sperry et al., 2007; Lazzarin et al., 2016; Echeverría et al., 2023). While vessel members typically measure only several hundred micrometers, tracheids can be exceptionally long in some species (e.g. some Araucariaceae species contain the longest tracheids among conifers; Carlquist, 2017). Longer tracheids generally enhance conductivity by reducing the number of cell-to-cell junctions, although pits continue to impose hydraulic resistance (Sperry and Hacke, 2004; 2005; Pittermann et al., 2005; Jacobsen, 2021).

Similar to xylem conduit dimensions, pit density and dimensions can be revealed via maceration in gymnosperms, angiosperms and in ferns (Burgess et al., 2006; Pittermann et al., 2006; 2010; 2011, Brodersen et al., 2014). Of these, the torus-margo pits are easiest to quantify via macerations and brightfield microscopy because diameters of both the pit and the pit aperture can be readily observed under higher magnification. However, pit attributes such as margo strand density, pit membrane thickness, and even pit membrane density (number of pits/wall area or pit membrane area per wall area) may be easier to quantify via scanning or electron microscopy (Pittermann et al., 2010; Brodersen et al., 2014). Nevertheless, xylem macerations are an important first step to identifying the characteristics of these and other attributes before one seeks these more sophisticated methods of conduit visualization.

In contrast to xylem, macerations have been much less frequently used in phloem research (Vilkovský and Čunderlík, 2017; Li et al., 2024) because being composed of living cells such as sieve tubes, phloem is much more delicate. However, after optimization, macerations can be used for the measurements of sieve cell and sieve tube element dimensions, similarly to xylem. For instance, Li et al. (2024) applied macerations using Franklin’s solution to determine dimensions of sieve tube elements and fibers in the phloem of 10 oak species. However, to our best knowledge, neither this nor other studies exploited macerations to examine the fine ultrastructure of sieve tube elements, such as dimensions and porosity of sieve plates or the coverage of the sieve cell surface by sieve areas. These structures have been previously observed mostly on cross or longitudinal sections using light, confocal or scanning electron microscopy (e.g., Jensen et al., 2012; Liesche et al., 2017; Knoblauch et al., 2020) as no protocol has yet been developed to allow detailed observations of these structures in macerated cells.

Developing a rapid maceration protocol to study phloem structure at the cellular and subcellular levels would streamline the research on phloem properties in the physiological, ecological, and evolutionary contexts. However, recent and detailed maceration protocols describing the maceration techniques for any of these tissues are practically missing, representing a limitation in the research of both tissues. The lack of precise protocols may be the reason why the method remains underutilized compared to conventional sectioning approaches used to study xylem and phloem (Liesche et al., 2017; Pace, 2019; Fonti et al., 2025).

Maceration techniques based on Jeffrey’s (Johansen, 1940) or Franklin’s solutions (Franklin, 1945) facilitate the separation of cells by degrading the pectic middle lamella, which normally binds adjacent cells together. Ideally, during the maceration, the cell walls remain typically intact, allowing anatomical study of individual cell types in three dimensions. One of the major obstacles in maceration can be attributed to the extraordinary anatomical diversity of xylem and phloem structure across plant species. The traditionally used maceration solutions may work universally, but the timings might be linked to factors such as wall chemistry and tissue density. Hence, it is important to accommodate this high degree of variation, especially traits such as different xylem composition and density, using appropriate and accessible maceration protocols.

Jeffrey’s solution has been traditionally and widely used for xylem maceration as it can rapidly break down plant tissues across a range of sample types from diverse plant species. However, its application poses several significant challenges that limit its practicality and safety. First, toxicity and environmental risks are major drawbacks of Jeffrey’s fluid. The chemical components (nitric acid and chromic acid at concentrations up to 10%) are hazardous to human health, requiring the implementation of strict safety protocols during handling and use (Shin et al., 2023). Its corrosive nature allows it to rapidly degrade various materials, including protective lab-coat fabrics. Disposal of Jeffrey’s fluid thus demands specialized waste management to minimize environmental contamination, increasing both labor and cost burdens for laboratories.

The other considerable disadvantage associated with the usage of Jeffrey’s solution is the need to precisely control the duration of the maceration. While Jeffrey’s solution is highly efficient at macerating samples, excessive exposure can destroy samples (long-term macerations can easily degrade individual cells by weakening their cell walls). Cells lacking secondary cell walls can be completely degraded when macerated with Jeffrey’s solution and the longest cells can often break. It is difficult to obtain accurate measurements of taxons where extended processing times are required for effective cell separation. Long tracheids also present technical challenges for anatomical study as the tracheids can often break during maceration with Jeffrey’s solution. As inaccurate and biased conduit length measurements result from cell breakage, researchers must carefully monitor exposure times, and adjust the exposure time to sample size and tissue type to avoid irreversible damage to the sample, including those of high scientific value. This makes the technique unreliable for long-term or high-volume studies that need consistent results.

An alternative to Jeffrey’s fluid is the use of acetic acid combined with hydrogen peroxide solution (hereafter Franklin’s solution; Franklin, 1945). First attempts to use acetic acid for maceration of xylem were done by Spearin and Isenberg (1947). Franklin’s solution offers several advantages, including lower toxicity and relatively easy waste management (neutralization requires only the addition of water and sodium bicarbonate). Furthermore, its use is also more cost-effective, as its reagents are readily available and affordable. Most importantly, Franklin’s solution leaves the integrity of the individual cells essentially intact, such that there is often little or no breakage of even the longest cells. These characteristics (low toxicity, cost-efficiency, and efficient maceration ability with no damage to xylem cells and a minimum damage to phloem cells) make Franklin’s solution an ideal alternative for researchers seeking a safer and more efficient method for maceration.

Previous studies modified the maceration technique based on the target species and research objectives without offering detailed protocols for practical use, as the focus was not on the maceration method itself (e.g. Xie et al., 2023; Gričar et al., 2024), or xylem and phloem were not the focus (e.g., Klahs et al., 2023). Some studies mention maceration protocols in books (e.g., Maiti et al., 2016; Ruzin, 1999; Sandoval, 2005), but these sources are often unknown or unavailable to many laboratories. This gap in clear, accessible protocols, as well as perhaps a lack of understanding of why macerations are essential for many accurate measurements, has limited the wider use of this technique, especially in fields like functional xylem biology and ecology, in which macerations have enormous potential to be applied to many different types of research.

Here we provide three detailed protocols designed to improve maceration techniques focusing on vascular tissues in woody species. By addressing the challenges mentioned above, we aim to contribute to the advancement of cellular research in xylem and phloem, making maceration technique more effective and widely used in both basic and applied research. We also aim to inspire expanded applications and foster new discoveries within the xylem and phloem research. The protocols differ in reagents and procedural conditions and address distinct practical considerations. Protocol 1 (P1) enables rapid maceration of xylem or phloem and facilitates quantitative analysis. Protocol 2 (P2) provides effective xylem maceration that have been proven in a wide range of species and wood densities, with intense high-contrast staining suitable for permanent slide preparation. Protocol 3 (P3) allows xylem maceration with extremely rapid dehydration and staining using minimal amounts of reagents, also producing high-quality permanent slides. These protocols aim to serve as flexible guidelines to enhance the quality of cellular analysis in xylem and phloem samples, ensuring that results are reliable, biologically accurate, and reproducible.

## 2 Materials and equipment

**Figure 1:**
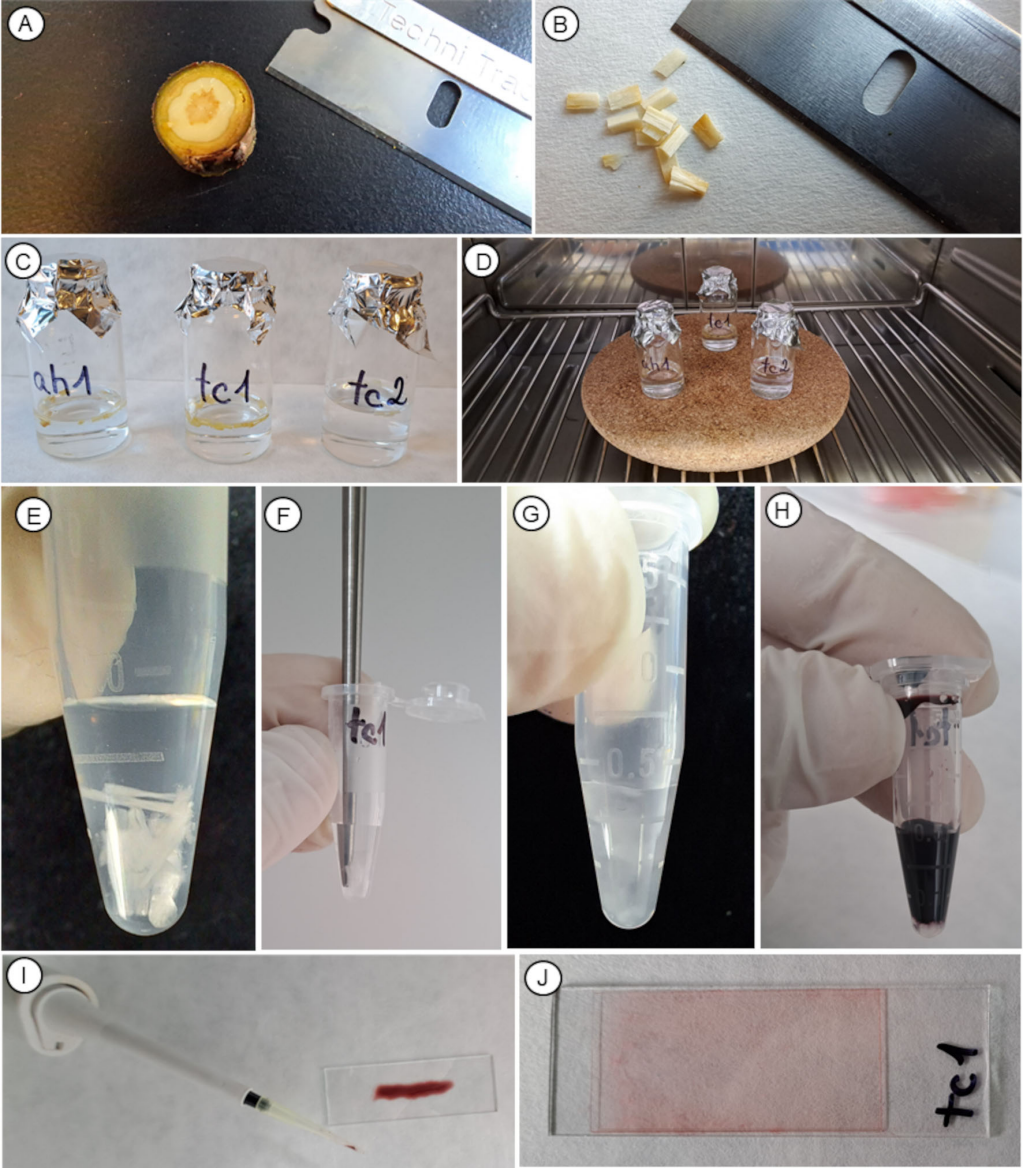
Photos showing the most important steps of the procedure of protocol P1. **(A)** Separating xylem or phloem tissue from the branch segment under a stereomicroscope using a sharp razor blade (step 2). **(B)** Cutting the separated xylem or phloem blocks into 1 mm-thick pieces (step 2). **(C)** Putting the pieces into a small vial filled with maceration solution and closing with a piece of aluminum foil (steps 3-4). **(D)** Placing the vials into the laboratory oven and heating them to 90-100 °C (step 5). **(E)** Example of an optimal amount of sample in an Eppendorf tube being homogenized (step 9). **(F)** Homogenizing the samples in an Eppendorf tube using an ultrasonic homogenizer (step 10). **(G)** The homogenization should result in milky cloudiness of the water in the Eppendorf tube (step 10). **(H)** Adding 200 μL of the staining solution and shaking properly (step 11). **(I)** Transferring 50 μL of the solution with homogenized and stained cells on a slide using a micropipette (step 13). **(J)** Final non-permanent slide that can be additionally sealed with nail polish.

**Figure 2:**
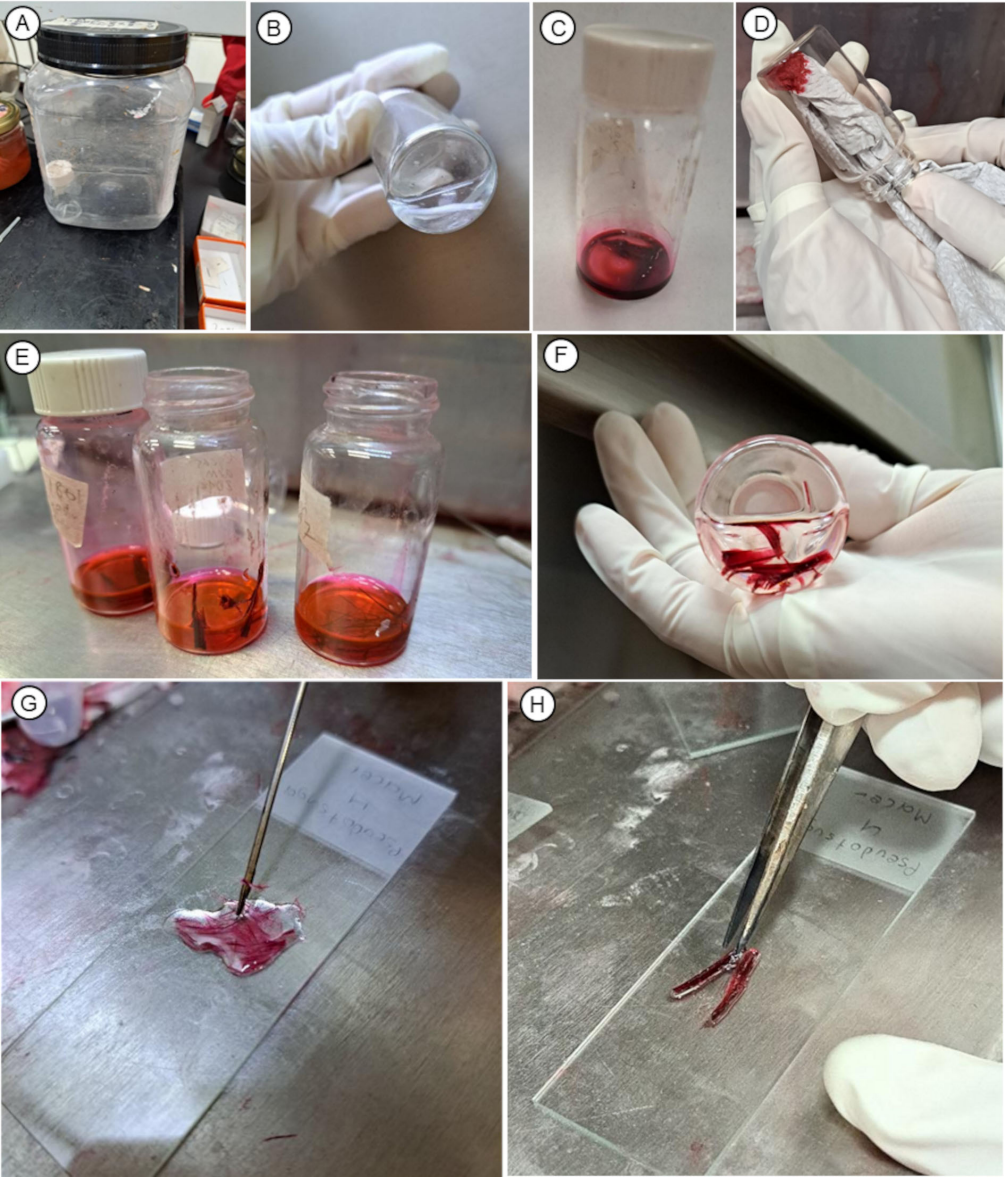
Photos showing general steps of the procedure of protocol P2. **(A)** Glass vial placed within a plastic jar positioned on an iron plate. **(B)** Tissue acquiring a white coloration prior to proceeding to the next step. **(C)** Saturated safranin solution; note the minimal volume required. **(D)** Excess stain removed using tissue paper as an alternative method. **(E)** Dilution of ethanol with clearing agent; additional clearing agent changes are still necessary. **(F)** Tissue intensely stained, softened, and ready for mounting. **(G, H)** Macerated tissue placed on a microscope slide.

**Figure 3:**
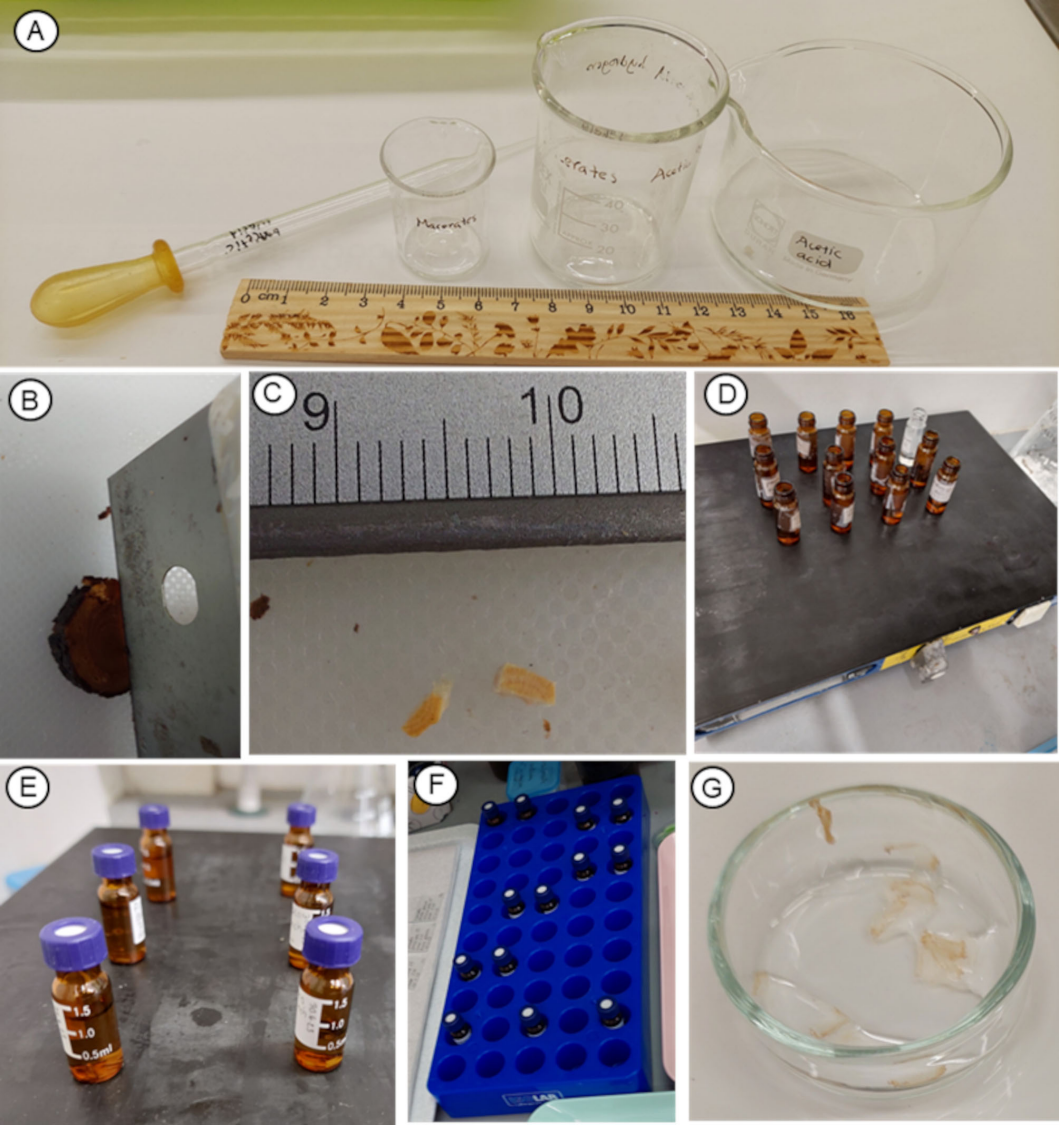
Photos showing the maceration and rinse steps of the procedure of protocol P3. **(A)** Material for preparation of Franklin's solution (step 1). **(B, C)** Cutting the xylem samples. **(D-F)** Vials on slide warming trace (step 3). **(G)** Samples after the third rinse (Step 5).

**Figure 4:**
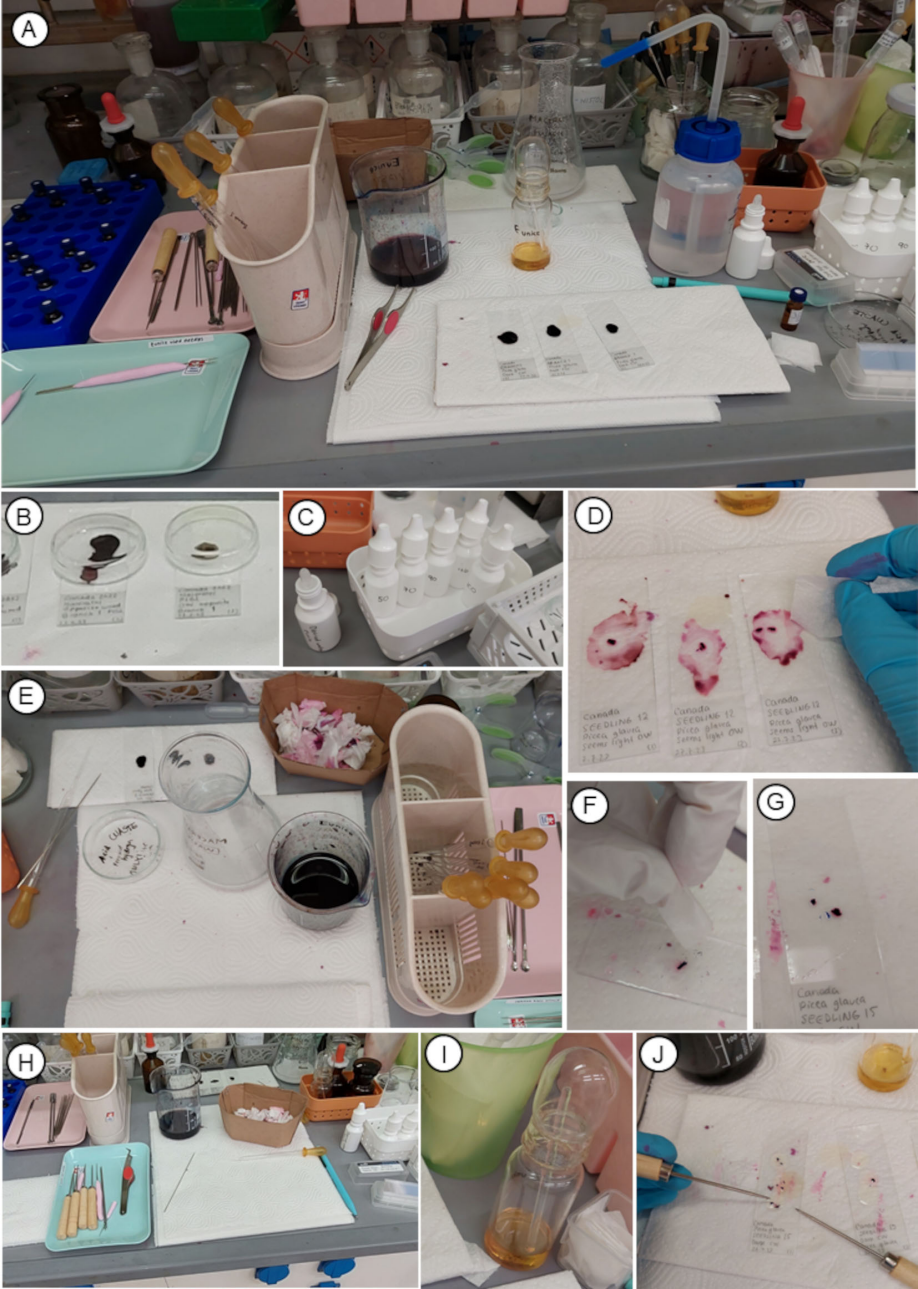
Photos showing steps of the staining and mounting of samples of the protocol P3. **(A, B)** Drops of stain applied (step 6). **(C)** Containers with ethanol and distilled water, **(D–G)** rinse and dehydration (step 7). **(H–J)** Permanent slides preparation (step 8).

In **Supplementary Table S1** we present an overview of each protocol’s materials, reagents, and equipment. Pictures of some examples of specific materials and equipment can be found in the figures corresponding to each protocol: P1, **Figure 1**; P2, **Figure 2**; P3 **Figures 3**, **4**.

## 3 Methods

Anatomical protocols are always just guidelines, and it is by no means necessary to follow them down to every detail. Laboratory techniques are evaluated based on their producing high-quality results in an acceptable time and at an acceptable cost in as safe a way as possible. Therefore, the protocols presented are simply to provide examples of the range of possibilities. Each laboratory can adapt them to suit their own needs.

The following text provides a description of three maceration protocols (overview in **Supplementary Table S2**; advantages and disadvantages in **Supplementary Table S3**), preceded by general considerations and notes. We highly recommend to consult and follow the *Safety and Hazard Considerations* section provided in the **Supplementary Material** prior to initiating any protocol.

### 3.1 Considerations for sample preparation prior to maceration

– We recommend that the sample to be macerated contains either xylem or phloem or only minimal proportions of other tissues. The presence of surrounding tissues reduces the proportions of the desired cells in the macerated sample, which complicates their proper identification and quantitative analysis. For macerations of xylem, we recommend removing all the bark and pith using a sharp razor blade, scalpel, or a razor scraper.
– In taxons and organs when applicable, separate phloem from xylem on the inner side and peripheral tissues (cortex and periderm) on the outer side.
– The choice of the specific xylem area from which the sample will be taken depends on the research question. To macerate a representative xylem sample (e.g. to account for variation between annual rings), we recommend cutting small slivers of xylem along the radial stem (or branch) direction, and 1 mm thick more or less. For spanning any radial variation, such as between earlywood and latewood, or across periodic bands of parenchyma, we recommend that samples for maceration measure about 1 × 1 cm viewed from the radial direction. Also, macerations can be made separately for contrast xylem sectors (e.g., imperforate tracheary element-bearing bands vs. parenchyma bands; earlywood vs. latewood) or isolated specific areas (e.g., narrow bands of vascular tracheids, reaction wood).
– To obtain representative phloem samples in juvenile stems, we recommend sampling the layer of secondary phloem next to the cambium to avoid mixing phloem with cortex cells.
– We recommend using a dissecting-stereoscopic microscope to cut smaller samples from a specific xylem area for more detailed analyses (e.g., specific annual rings in the xylem) or to isolate phloem from surrounding tissues. If a dissecting microscope is not available, a digital microscopic camera or a magnifying glass (e.g., used by watchmakers, for jewelry) may also be used instead.
– Ensure the pieces of xylem or phloem are as thin as possible to allow full penetration of reactants. Thicker samples prolong maceration. We recommend that the samples should not be thicker than 1–2 mm.
– To account for potential variability among samples and safeguard against the potential loss of slides during preparation or handling, it is recommended to prepare duplicates or multiple slides.

### 3.2 Considerations for preparing Franklin’s solution

– The proportions of hydrogen peroxide and acetic acid can be adjusted as necessary. The solution can even be prepared without needing to measure carefully the proportion.
– If the proportion of hydrogen peroxide is increased, the solution becomes more oxidizing and less acidic. This accelerates the breakdown of lignin and other organic matter but can also over-bleach or damage the cell walls, especially in delicate tissues. The reaction will be more vigorous, sometimes producing heat and bubbles that can fragment the material, which can actually help sometimes because the cells separate on their own.
– If the proportion of acetic acid increases, the mixture becomes less oxidizing and more acidic. This slows the reaction and can help preserve the integrity of the cell walls, leading to cleaner separations with less cell wall erosion, but it also prolongs the maceration time. Too much acid, however, can begin to hydrolyze cellulose and soften or distort the walls.
– Franklin’s solution waste can be poured into a large beaker (or glass container) filled with water and sodium bicarbonate (acts as a neutralizing agent).

### 3.3 Staining of macerated cells

– Ensure intense staining to enhance contrast of the macerated cells. Multiple dyes provide good staining results for cells possessing secondary lignified cell walls (imperforate tracheary elements, vessel members), while a relatively limited number of dyes can bind to the cells having typically cellulosic primary walls (typically sieve tube elements). Hence, contrast staining of macerated phloem cell types is typically more challenging than that of xylem.
– For P1, we recommend using stains at concentrations ranging from 0.1% to 1% (w/v; weight per volume) depending on the tissue type and desired staining intensity.
– Recommended stains for xylem maceration include: i) Safranin dissolved in water or 50% ethanol: stains lignified cell walls into pink or red; ii) Fast green or Astra blue dissolved in water: color cellulosic primary cell walls into green or blue color, therefore highlighting less lignified tissues and non-lignified ones (although in maceration may not provide consistent results); iii) Toluidine blue dissolved in water: stains both lignified and cellulosic cell walls into green to blue color; iv) Congo red: stains cellulosic cell walls into red color.
– Recommended stains for phloem maceration include: i) Astra blue: stains cellulosic primary cell walls into blue color, ii) Congo red dissolved in water: stains cellulosic cell walls red.
– Using contrast-enhancing dyes is useful for highlighting different cellular components and facilitating detailed analysis of cell structure. However, maceration degrades some cell components in phloem (e.g., callose in sieve tube members and sieve cells) and alters the cell wall properties. Therefore, several stains commonly used to stain sections may not stain macerated phloem cells, or their use may not provide consistent results.
– In P2, safranin is used saturated, and pinches of the counterstain can be added later if necessary.
– In P3 liquid safranin and liquid Astra blue are simply mixed in a 1:1 solution.

### 3.4 Warning to avoid cross-contamination

– Be aware to use clean tools while working with the macerated tissues. For example, use clean tweezers, dissecting needles, or similar tools to divide the macerated tissue evenly across slides. Residues of one maceration stuck to the tweezers or needles, which can unintentionally import cells from a previous sample or preparation, which could lead to erroneous conclusions.
– To avoid cross-contamination between samples it is important to be aware of the clearing agent used. For protocol P2, keep the clearing agent far away from any source of water, clean the tools using flame. For protocol P3 we suggest keeping a container with sufficient distilled water nearby to rinse the tweezers between each sample, and dry them using separate clean paper after each rinse.
– Do not recycle the old maceration solution, as the hydrogen peroxide degrades during boiling (losing its effectiveness) and the solution can be contaminated with cells, for example, from a different species, sample, or tissue section.

### 3.5 Safety and hazard considerations

– For preparing Franklin’s solution, it is highly recommended to wear protective gear such as gloves, a lab coat, and goggles, and to work in a fume hood; protocols P2 and P3 include alternatives in case a fume hood is not available. We also recommend using glass pipettes and glass containers to prepare the solution.
– Always have bicarbonate nearby when working with acid.
– Franklin’s solution produces gases that can build up pressure inside sealed containers, even at room temperature. Therefore, it is very important not to screw the lids tightly, allowing for the hydrogen gas that bubbles out of the tissue during the maceration process to escape; otherwise it can build up pressure and the vial can explode spattering acid all and glass (explosions have occurred even at room temperature and at 40 °C). If the lids are tightly closed, this pressure may cause the containers to burst, even if they are made of glass. For this reason, instead of using sealed vials, aluminum foil or funnels can be used to minimize loss of the solution during heating (as in P1). Another option is placing the sample containers inside a larger container (as in P2), or using vials with a special cap (as in P3). Placing baking soda (sodium bicarbonate) around them to help neutralize any potential spills or leaks is recommended for all maceration protocols.
– A laboratory oven can be used for heating during the maceration process (as in protocol P1); however, to prevent the oven from being exposed to acid vapors from Franklin’s solution, we recommend using a slide warming tray (as in P2 and P3). A hot plate can be used instead, although it has some disadvantages: it is difficult to control the temperature without a digital thermometer, and it may easily happen to exceed the boiling point. Thus, it is advisable to slowly and continuously increase the temperature of the hot plate, then maintain it slightly below the boiling point.
– Be aware of the jar or container composition used while heating. For example, polypropylene (PP) can withstand temperatures up to 120 °C. A classical polyethylene terephthalate (PET) starts deform around 70 °C.
– Congo red is a universal stain that can be used for staining both xylem and phloem with very good contrast for all xylem and phloem cell types, including the hardly stainable sieve tube members in phloem. In contrast to Astra blue, which can be observed only in bright field, the cells stained with Congo red can be observed using light, epifluorescence, and a confocal laser microscope. Importantly, Congo red, similarly to other benzidine-derived dyes, is toxic and carcinogenic. Its usage thus requires adhering to the strict rules in using protective gear and waste management. While handling the stain, staining the samples, or following manipulation with the microscopic slides, it is necessary to wear goggles, gloves with suitable chemical protection, and a lab coat to prevent direct contact of the stain solution with the skin. Do not empty the used stain or macerated samples into the drain. Always dispose of the samples used and slides into hazardous waste.
– Do not empty any used stain or clearing agent into the drain. We recommend collecting all reagent waste in clean glass containers (e.g., recycled juice jars) and storing them securely until appropriate disposal. We recommend neutralizing Franklin’s solution waste with sodium bicarbonate, and placing it in separate containers from other types of waste (e.g. stains and clearing agents).
– During and after mounting permanent samples within protocols P2 and P3, it is strongly recommended to minimize inhalation exposure by ensuring proper ventilation. Allowing the slides to fully dry before storing them is highly recommended due to the slow-polymerizing nature of the mounting resin used in both protocols.
– Particular warnings concerning specific mounting medium used in protocol P3, and the optional clearing agent: the mounting medium used in P3, both liquid and the vapors, are highly toxic and flammable. Ventilate always when using it. Use nitrile gloves. The optional clearing agent recommended in P3 consists of orange terpenes, being a safer alternative to, for example, xylene; however, it dissolves plastic. Therefore, we recommend using glass tools when working with this clearing agent (containers, pipettes, etc.), and waste must not be emptied into the drain. Appropriate ventilation is still required because it is flammable.

### 3.6 General notes for selecting a suitable protocol

– A comparative overview of protocols P1, P2, and P3 is presented in **Supplementary Tables S1**–**S3**: **Supplementary Table S1** details materials and equipment, **Supplementary Table S2** summarizes objectives, methods, and typical times for maceration and slide preparation, and **Supplementary Table S3** highlights advantages and disadvantages.
– P1 is optimized for the maceration of both xylem and phloem tissues. The protocol P1 partly originates from a traditionally used original protocol (OP) used for maceration of xylem. OP is presented in **Supplementary Material** and in **Supplementary Figures S1**–**S5**. Both protocols (OP and P1) allow the rapid preparation of non-permanent and semi-permanent slides.
– Protocols P2 and P3 are designed for xylem maceration and the preparation of permanent slides.

#### 3.6.1 Protocol P1

Steps (1-15) are the following:

1. Prepare a solution consisting of one part of fresh hydrogen peroxide (35% H_2_O_2_) one part of glacial (i.e., absolute, 100%) acetic acid in a glass flask. We suggest using a 1:1 ratio of glacial acetic acid and 35% hydrogen peroxide to accelerate the maceration procedure. However, it is possible to use a more diluted solution (for example, glacial acetic acid:35% hydrogen peroxide: water in a 1:1:1 ratio).

2. Cut the separate xylem or phloem block into small pieces (1 mm thick) using a sharp razor blade or a scalpel (**Figures 1A, B**).

3. Put the cut pieces of samples into the small glass vial (20 mL) or into the small Erlenmeyer flask (25–50 mL) and add approx. 5–10 mL of the maceration solution.

4. Close the vial with a piece of aluminum foil to minimize the loss of maceration solution during sample heating by evaporation (**Figure 1C**). Alternatively, a small funnel can be used instead of foil. Avoid closing the vials tightly with a cup to minimize the risk of explosion heating.

5. Place the vial into a laboratory oven set to 90 – 100 °C (**Figure 1D**) and heat the samples for 2–6 hours, depending on the species and sample type. For easier manipulation with multiple hot vials, place them on a cork pad. Alternatively, place the vial on a hot plate and maintain the temperature of the solution slightly below 100 °C to avoid ebullition. Maceration can be performed even at lower temperatures (50-70 °C), but the entire process typically lasts significantly longer.

6. We recommend proper testing of the duration of maceration with respect to the sample identity. For this purpose, put a piece of the macerating sample every hour onto a microscopic slide and try to gently separate the cells with a pair of needles. Macerated samples have a white color, and the cells can be easily separated even after gently touching the sample with a needle. Maceration for 3–4 hours, and 2–3 hours is typically sufficient for separating individual xylem and phloem cells in various angiosperm and gymnosperm tree species, respectively. When macerating delicate tissues composed mostly of non-lignified cells, such as phloem, avoid prolonging the duration of maceration to minimize the risk of damage to these cells.

7. When the maceration is done, remove the vials with the cork pad from the oven and let them cool down for 10 min. When heating samples on the hot plate, use tongs or heat-resistant gloves to manipulate the hot vials.

8. Subsequently, the samples should be thoroughly washed in water to stop the maceration. Replace the maceration solution with distilled water several times until it no longer smells of acid. To minimize the risk of losing samples, use a glass dropping pipette with a rubber bulb to remove the maceration solution. Alternatively, pour the solution with macerated samples into a small beaker and gently transfer the samples into a cell strainer floating in water (e.g., **Supplementary Figure S3C**). Wash the samples repeatedly by submerging the strainer with samples in fresh distilled water until they no longer smell of acid. Properly washed samples can be stored in water in a fridge for several days.

9. Pipette 200 μL of distilled water into an Eppendorf tube (we recommend using 1.5 ml tubes with a conical bottom). Use a pencil brush or tweezers to place a macerated sample into the tube. The transferred samples should occupy approximately one-fourth to one-third of the water volume (**Figure 1E**). The amount of sample put into the tube corresponds to the number of cells transferred to the microscopic slide. Reducing the amount of macerated plant material decreases the number of overlapping cells and facilitates their observation, but it simultaneously decreases their total number on the slide.

Note: a simple Pasteur pipettes can be used instead of a micropipette. The volumes represent a rather general recommendation and the effect on homogenization and staining is really marginal if slightly more or less water or stain is added. The micropipette is mostly useful for the transfer of the homogenized cells on the slide in the precise volume, as it accelerates the whole process of the slide preparation and improves its quality, as there is no need to remove excess water and loose cells from the slide. By using a micropipette, cross-contamination is avoided, as a new pipette tip for each sample is used. The other advantage of the micropipette is in controlling the amount of the macerated cells that will be transferred on the slide by creating a larger or smaller hole in the pipette tip.

10. For optimal homogenization of the macerated samples, we highly recommend using an ultrasonic homogenizer equipped with a 1 mm thick sonotrode (**Figure 1F**). Homogenize the samples in the Eppendorf tube by applying 2–3 cycles of longer duration (set to 0.7-0.9) and medium amplitude (set to 50-60%). Such a technique allows gentle separation of individual cells and minimizes their damage or deformation (e.g., bending or twisting of fibers, tracheids, sieve tube members, or sieve cells). Homogenization of properly macerated samples should result in milky cloudiness of water in the Eppendorf tube (**Figure 1G**). If the samples cannot be easily homogenized, prolong the maceration duration. Avoid using homogenization for a significantly longer period to avoid cell damage or deformation (namely, long fibers or non-lignified phloem conduits are sensitive to damage when over-macerated). Homogenization with the ultrasonic homogenizer is important to properly separate sieve tube elements in phloem from other cell types to be observed in sufficient numbers. If an ultrasonic homogenizer is not available, samples can be homogenized manually by shaking the Eppendorf tube. However, additional manual homogenization of the sample on the slide is usually required.

11. After homogenization of the sample, add 200 μL of the selected stain (recommended 0.5%; for more details see staining section above) into the Eppendorf tube and shake the tube properly to facilitate a good contact of the stain solution with all homogenized cells. Let the samples stain for at least 5 min (**Figure 1H**).

12. Subsequently, dilute the solution by adding 600-800 µL of distilled water and shake the tube properly by hand or using a vortex mixer. The stained samples can then be used for the preparation of microscopic slides (see below) or stored in a fridge for several days. To prepare semi-permanent slides, it is possible to add 600-800 µL of glycerin instead of water and properly mix.

13. Cut 1–2 mm of the yellow pipette tip (for volumes < 200 µL) with a sharp razor blade to enlarge the pipette opening and be able to easily suck in the homogenized samples. Use the adjusted tip and automatic pipette to transfer 50 µL of the homogenized and stained sample to the microscopic slide. Spread the sample over the larger area of the slide (**Figure 1I**). If larger cell clusters are present, separate them gently with the pipette tip or a needle over the slide. Alternatively, raise and lower the coverslip repeatedly to further loosen the grouped cells. Slowly lay down the coverslip using the needle to squeeze out all the bubbles from one side to the other to avoid bubble formation. Avoid removing an excess solution with the redundant water using a laboratory wipe or a piece of filter paper to minimize loss of macerated cells from the slide. Also, avoid pushing on the slide to prevent the destruction of fragile cell types, such as sieve tube elements. The applied volume of 50 μL is optimal for the rectangular cover slips 24 x 50 mm and does not typically require removing redundant solution.

14. Cells in an optimally prepared sample are homogeneously distributed across the whole slide area (**Figure 1J**).

15. We recommend using nail polish to seal the edges of the cover slip to prevent water leakage during manipulation with the slide and to avoid water evaporation. The slides with water can be stored for several days. We recommend storing the semi-permanent slides in a folder to prevent cell movement and minimize the risk of water leakage.

#### 3.6.2 Protocol P2

Steps (1-20; general steps in **Figure 2**) are the following:

1. Place the xylem slivers in a small vial containing Franklin’s solution (e.g. 1:1) of hydrogen peroxide (30% H_2_O_2_) and glacial acetic acid (e.g. 1:1; proportions can vary, see section above).

2. Cover the xylem with the solution, leaving ample air in the vial. Clear glass scintillation vials with caps are very convenient as they are transparent glass and it’s easy to observe the progression of the sample. Their size is ideal (20 mL), and their resistant materials mean that they can be used throughout the process, from maceration to dehydration to staining and mounting. They are reusable, which mitigates their relatively high cost. Leave the xylem in the solution for at least 24 hours, do not screw the lids tightly (**Figure 2A**).

3. The vials can be put inside a closed plastic jar or box (to keep fumes and liquid inside in the case of an accidental explosion) on a slide warming tray (approx. at 45°C), ideally inside a fume hood.

4. The solution will emit acetic gas. If a fume hood is not available, to contain the fumes and any explosion, keep the vials in a larger container (container is taller than the glass vials).

5. You can leave the same solution macerating until the tissue is soft; as long as bubbles are coming out, the reaction is proceeding. When opening the large jar, using gloves is highly recommended because when the lids are open slightly, the large jar fills with acid fumes that condenses on the walls. If the tissue is taking more than a day or two to macerate, the solution can be replaced. When the tissue appears slightly translucent, often entirely white, and does not resist piercing with a needle, proceed to rinse and dehydrate it. The tissue typically hardens slightly during the dehydration and staining process, so making sure it is quite soft, repeating cycles in the solution, if necessary or simply leaving it until it reaches the desired softness, is helpful.

6. After discarding the used Franklin’s solution, rinse by gently filling the vial completely with water. Rinse the cap as well, as it may retain some solution. Fill the vial slowly from the tap to avoid disintegrating the xylem piece. Discard the water immediately or after approximately 10 minutes. We do not recommend reusing Franklin’s solution due to the small volumes involved and the risk of cross-contamination with cells from different samples. Note that a few cells might be lost but the tissue remains in a coherent piece that will be separated during the mounting process. However, we recommend to be very gentle when discarding solutions and reagents.

7. Refill the vial with water and leave it on the slide warming tray for 1–3 hours. Discard the water and repeat the process until there is little to no smell of acetic acid. Appropriate rinsing prevents further degradation. Franklin solution continues to be rinsed with distilled water, usually once or twice is all that is needed; in any case, in the subsequent dehydration steps the rinsing process continues. To speed up the process, a small amount of baking soda can be added to neutralize the acid, but this may cause bubbles that could damage fragile tissues.

8. To remove liquid, use an intact pipette. To add liquid, the fine tip of a plastic pipette can be cut off, creating a wider opening that produces a gentler stream, thereby preventing loss of tissue. Gently fill the vial:

o First with 70% ethanol, and place it on a slide warming tray for 24 hours.

o Then with 96% ethanol, and place it on a slide warming tray for 24 hours.

We recommend using abundant amounts of 70% and 96% ethanol (e.g. fill the vial) to help continue rinsing.

9. Barely cover the tissue with 100% ethanol and place it on the slide warming tray for 3 hours.

10. Perform three changes of pure ethanol (absolute or near 100%; hereafter 100%) in total. For 100% ethanol we recommend to use just enough to cover the tissue. Changes might be as follows:

o 100% ethanol for minimum 3 hours, or 12–24 hours.

o 100% ethanol for minimum 3 hours, or 12–24 hours.

o 100% ethanol for minimum 3 hours or 12–24 hours; overnight (ideally 24 hours).

Two of those three changes last 3 hours and one is at least overnight (ideally 24 hours). The order of these changes is not important, as long as one of them is about 12–24 hours.

Ethanol with 96% and 70% concentrations is usually cheaper, whereas absolute ethanol (near 100%) is usually more expensive, hence the differences in volumes used during the dehydration steps (and because large amounts are unnecessary for effective dehydration and because the tissue has by now been rinsed of Franklin’s).

11. Once the dehydration process is completed, cover the tissue with a saturated solution of safranin in 100% ethanol. It is not necessary to use precise concentrations of safranin since it is practically impossible to over-stain with safranin. Since safranin is poorly soluble in 100% alcohol, simply add safranin powder to 100% ethanol until a small sediment remains at the bottom. To ensure intense staining, we stain for at least 24 hours, but typically for several days.

12. Once stained, retrieve the safranin with a plastic pipette. It can be reused for other staining processes. We recommend filtering it to avoid cross-contamination of tissues.

13. Gently rinse the vial with 100% alcohol to remove the excess safranin (this rinse can be deposited in an open jar; the alcohol evaporates, concentrating the safranin, which can then be recovered). This step requires working quickly to minimize destaining; this step is just a rinse because the tissue is already dehydrated. After this quick 100% ethanol rinse, the alcohol is quickly pipetted off or poured off. After the rinse, insert a paper towel into the vial under some pressure to remove rinseate from the vial walls and to draw alcohol from the samples (**Figure 2D**). The objective is to remove the ethanol without destaining.

14. In the hood, fill the vial with the clearing agent (the solution will turn pink, as residual alcohol continues to strip the tissue; see details of reagents used in **Supplementary material**). The goal is to dilute the ethanol as quickly as possible and replace it with the clearing agent because the alcohol dilutes the safranin, diminishing the stain, while the clearing agent dilutes the alcohol. Discard the clearing agent after approximately 5–10 minutes.

Tissue can be also stored in the clearing agent until mounting.

15. Perform 2–3 changes of clearing agent (or more if the liquid continues to turn pink). You can leave the tissue for 24 hours between changes to be thorough, but often a duration of 10–15 minutes between changes is sufficient. The clearing agent should remain perfectly clear.

16. At the end of the process, the tissue will be intensely stained, very soft, and ready for mounting (**Figure 2F**). It can be stored in the clearing agent in a scintillation vial until mounting, at least for weeks, but keep an eye on the level of the clearing agent. If it evaporates, it is very hard to re-infiltrate the tissue with a clearing agent and it is generally necessary to re-hydrate or even re-macerate.

17. Apply generous drops of mounting resin on each slide. Add a few drops of xylene from the vial to prevent the resin from drying out, especially if the resin is not very liquid.

18. Place a piece of the softened tissue on the slide. Using a needle, gently remove cells either from one edge of the tissue piece or by gently scraping the widest surface (ensuring everything is cushioned with plenty of resin). Giving a gentle tapping to the tissue is often enough to fully macerate it. Often you can use the viscosity of the resin to macerate the tissue gently, simply by moving the needle up and down in the resin above the tissue. This helps avoid breaking cells.

19. Once you have the desired number of cells on the first slide, move the remaining tissue to the next slide (**Figure 2H**).

20. Place a cover slip on the slide, adding a few drops of clearing agent if necessary. Since the mounting resin used shrinks significantly when dry, especially with heat, we recommend allowing the slides to dry (polymerization of mounting resin) at room temperature inside a fume hood.

#### 3.6.3 Protocol P3

To provide a general idea of the materials and setup, itemized materials and reagents used in P3 can be found in https://zenodo.org/records/18198262). Steps of Protocol P3 (1-9) are the following:

1. Prepare a fresh 1:1 solution of fresh hydrogen peroxide (35% H_2_O_2_) and glacial acetic acid in a small glass beaker, depending on the total volume to be used (**Figure 3A**).

2. Cut each xylem piece (**Figures 3B, C**) and place it inside a separate crystal vial, ensuring one piece per vial. We recommend small vials equipped with special caps that feature a hole sealed by a flexible silicone insert. These caps can be loosely closed to help contain gases when the fume hood is off, while still allowing some ventilation. The silicone insert also minimizes solution evaporation during heating on a hot plate and helps prevent spills. If necessary, the silicone can be punctured with a sewing needle to allow gas release. Because the silicone is flexible, it can expand slightly to relieve pressure buildup, reducing the risk of vial breakage during boiling or heating. Yet, never close vials tightly.

3. Place the vials on a slide warming tray, without cap, inside a fume hood to heat the solution overnight or for 24 hours (**Figures 3D, E**). If heating is not possible, vials with caps can be placed in racks or holders to keep them upright and stable (**Figure 3F**) and leave the samples in the solution, at room temperature, for approximately one month. Depending on the sample and the duration of exposure, the solution may be changed once, twice, or not at all.

4. Inside the vial, rinse with distilled water twice the sample pieces (macerated tissue). Use a different glass pipette for each vial. It is not necessary to wait between rinses.

5. Transfer the samples with the third rinse (with distilled water) to a small Petri dish (**Figure 3G**).

6. Prepare a solution 1:1 of liquid safranin + liquid Astra blue (particularly, pH 2.5, for mucin detection was used for this protocol). Apply 1 to 3 drops of safranin-Astra blue stain directly onto the slide with the macerated tissue (**Figure 4A**). Let it sit for 1 to 15 minutes (or longer, if necessary). A clean Petri dish can be used to cover the slides with stain droplets, helping to prevent stain evaporation (**Figure 4B**).

7. Rinse the stain by sequentially applying 1 to 5 drops of the following solutions (**Figures 4C–G**) directly onto the slide and removing each immediately using first a pipette and then an absorbent paper:

o Distilled water.

o 50% ethanol.

o 70% ethanol.

o 90% ethanol.

o 100% ethanol (this 100% ethanol rinse is recommended to be done as fast as possible to avoid loss of stains). Use a separate, clean pipette and paper (**Figure 4E**) for each concentration to avoid cross-contamination.

8. Immediately add a few drops of mounting resin (ideally compatible to a certain volume of water) to the first slide and gently separate the tissue using dissecting needles (**Figures 4H–J**). Repeat this step with the second and third slides as soon as possible. It is recommended to carefully place the coverslip immediately after mounting. Ideal size of coverslip for macerations is 24 x 50 mm. Use clean tools (*e.g.*, dissecting needles) to avoid cross-contamination.

In this protocol, a clearing agent is not needed. Yet, optionally, between the 100% ethanol rinse and applying mounting resin, some drops of a clearing agent, compatible with the mounting resin, can be applied.

9. Place the slides in an oven at 65 °C. Usually the liquid mounting resin in the slides becomes solid (polymerized) enough for observation with a microscope after one night to 3 days in the oven, or approx. one week at room temperature inside a fume hood. Three months in an oven at 65 °C is a standard time recommended to ensure the complete polymerization of the mounting resin used in this protocol, for safe-storing of the slides.

## 4 Results

**Figure 5:**
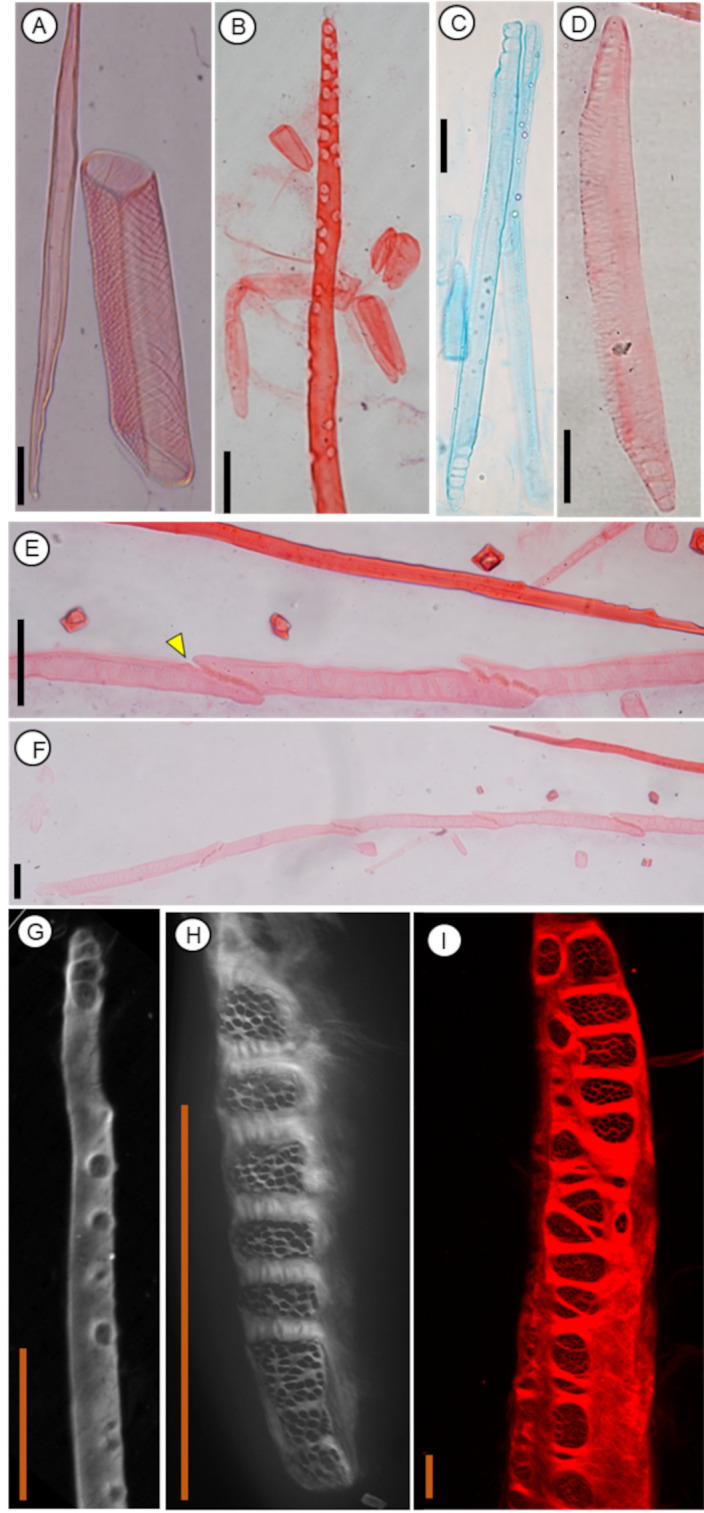
Anatomical features of macerated xylem and phloem cells of a gymnosperm and angiosperms using protocol P1 and stained with Congo red dye (except C); Scale bar = 50 µm. **(A)** A xylem vessel member (right) and a libriform fiber (left) in *Tilia cordata* and observed in bright field. **(B)** End of a phloem sieve cell in *Picea abies* observed in bright field. **(C)** A phloem sieve tube member in *Tilia cordata* stained with Astra blue and observed in bright field. **(D)** A phloem sieve tube member in *Tilia cordata* observed in bright field. **(E)** and **(F)** Connected phloem sieve tube members in *Quercus petraea* observed in bright field. **(G)** End of a phloem sieve cell in *Picea abies* observed with a confocal microscope. **(H)** A detail of a sieve plate in a sieve tube element of *Carpinus betulus* observed with a confocal microscope. **(I)***Carpinus betulus*, observed with a confocal microscope, scale bar = 10 µm; the sieve tube element was stained by Congo red. Images G and H are shown in greyscale, while false red color is used in image I.

**Figure 6:**
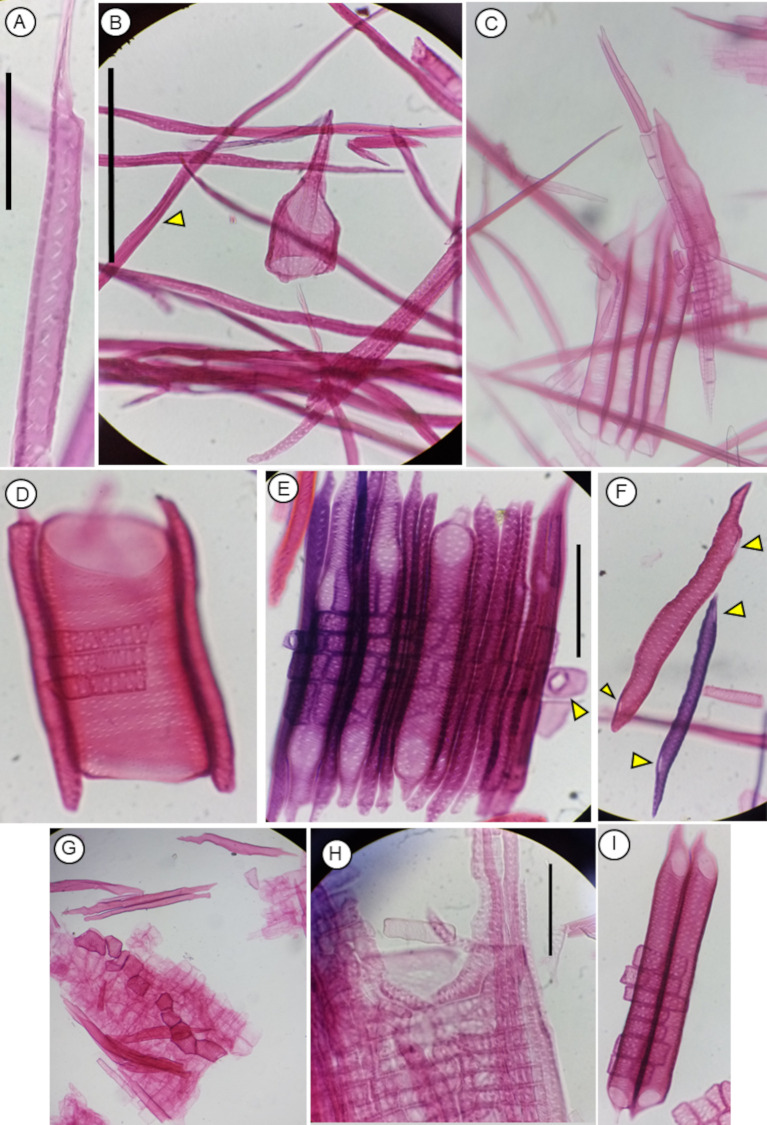
Anatomical features of angiosperm cells observed under the microscope after maceration using protocol P2, stained with safranin. Scale bars = 100 µm. **(A)***Austrobaileia scandens* true tracheid. **(B)***Larrea tridentata* vessel element and true tracheids. **(C)***Licaria nayaritensis* vessel elements, fibers, radial parenchyma. **(D)***Ziziphus parryi* vessel elements of similar length as tracheids. **(E)***Ziziphus parryi* vasicentric tracheids, the arrowhead shows radial parenchyma; pit in radial parenchyma can be observed at the right. **(F)***Ziziphus parryi* vessel elements that seem tracheid but macerations reveal it has opposite perforation plates. **(G)***Pachypodium lealii* vessel elements and a piece of vessel showing approximate architecture, radial parenchyma, fibers. **(H)***Quercus berberidifolia* vasicentric tracheids radial with the shape of the radial parenchyma, and a surrounding earlywood vessel element. **(I)***Ziziphus parryi* vessel elements and radial parenchyma.

**Figure 7:**
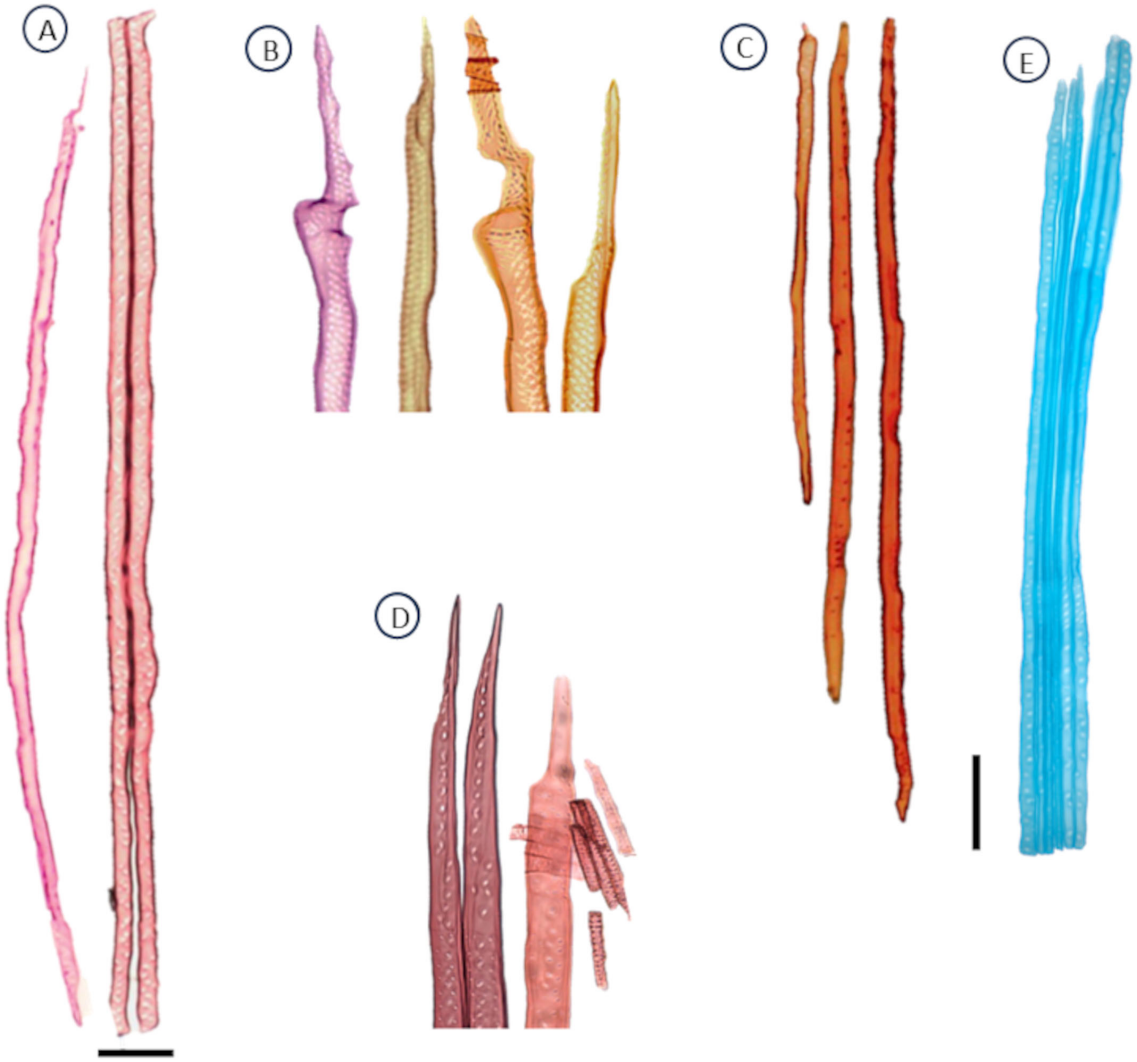
Anatomical characteristics of the tracheids of gymnosperms, observed under the microscope after maceration using protocol P2; **(A)***Retrophillum minus*. **(B)** A cycad, *Dioon* sp*inulosun* tracheid endings. **(C)***Widringtonia nodiflora* tracheids from the same individual. **(D)***Abies alba*. **(E)***Picea glauca* tracheids with similar length and different diameters, scale = 100 µm. **(A–D)** protocol P2 was used; staining: safranin. **(E)** correspond to Protocol P3; staining: safranin + astra blue. Scale bar = 100 µm.

We presented updated xylem and phloem maceration protocols using Franklin’s solution, for preparing both permanent and non-permanent sample slides. These protocols can be used to study xylem and phloem structure in a wide range of woody specimens (with different lignification degrees, and not necessarily with secondary xylem), including gymnosperms and angiosperms. Here, we also demonstrate that optimized maceration protocols allow for the detailed study of individual xylem or phloem cells of potentially all woody species. Below, we present **Figures 5**–**7**, illustrating some examples of the results that can be observed after using each maceration protocol. Macerated cells can be used for general quantitative determination of cell dimensions and morphology as well as to study cell ultrastructure in detail (e.g., structure perforation plates, sieve plates or pits).

*Taxonomic range of proven applicability for each protocol*.

By applying P1, the homogenized cells are evenly distributed across the slide with minimal damage, facilitating their identification and quantitative analysis. Staining with the aforementioned dyes enhances the contrast of all xylem and phloem cell types. In phloem, staining with both Astra blue and Congo red stains all cells with a slightly lower contrast for sieve tube elements compared to the lignified cells. While staining of phloem with Astra blue allows observation of the macerated cells in bright field, the cells stained with Congo red can be observed using a light, epifluorescence, or confocal laser microscope. Namely, the confocal laser microscope allows 3D reconstruction of the whole macerated cell or observation of cell ultrastructure, such as sieve pores in sieve plates or sieve areas, with great detail.

Protocol P1 is an optimized version of the original protocol (OP, see section above in Methods: General notes for selecting a suitable protocol), and these protocols have been successfully used for branches of conifers (e.g. *Abies alba*, *Metasequoia glyptostroboides, Picea abies, Pinus sylvestris, Pseudotsuga menziesii*), as well as diffuse- (e.g., *Acer platanoides*, *Carpinus betulus, Corylus avelana, Crataegus monogyna, Fagus sylvatica*, *Malus domestica, Prunus avium, Tilia cordata*) and ring-porous angiosperm tree species (*Quercus robur, Robinia pseudoacacia*). Some examples of results using the protocol P1 are shown in **Figure 5** for the optimized protocol P1, and for the original protocol OP in **Supplementary Figures S6**–**S10**.

**Figure 8:**
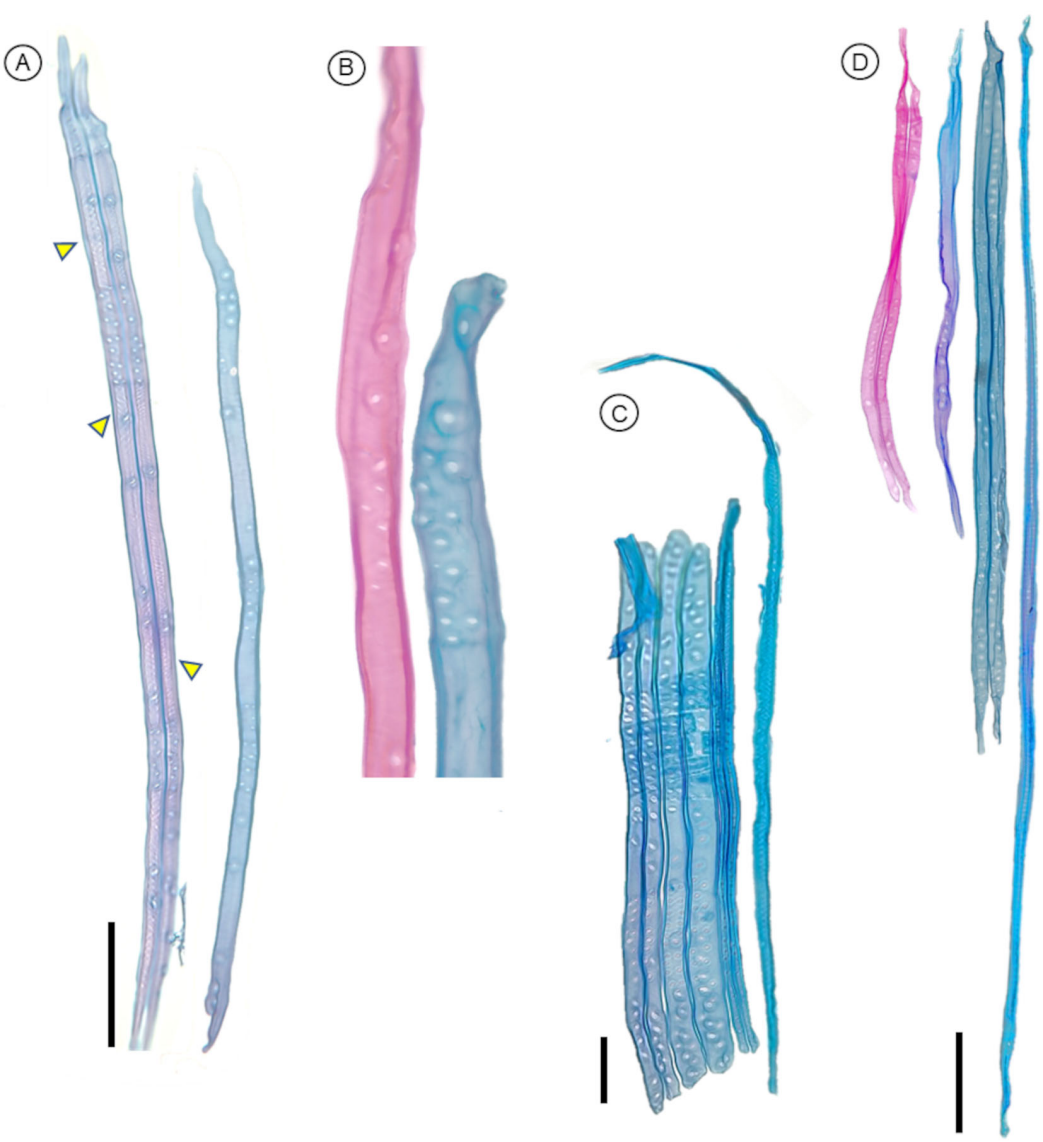
Anatomical characteristics of tracheids in gymnosperm species that can be observed after using the maceration protocol P3, and stained with safranin and Astra blue. **(A)***Picea abies* tracheids showing the distribution of pits and different pit types connecting radial parenchyma or other tracheids; helicoidal thickenings are also visible (arrowheads); scale bar = 100 µm. **(B)** Close-up view of pits in tracheids with contrasting staining. **(C)***Picea glauca* tracheids with different diameters and lengths observed in the same section; the three tracheids on the right show helicoidal thickenings; scale bar = 200 µm. **(D)***Larix decidua* tracheids with different lengths; scale bar = 100 µm.

**Figure 9:**
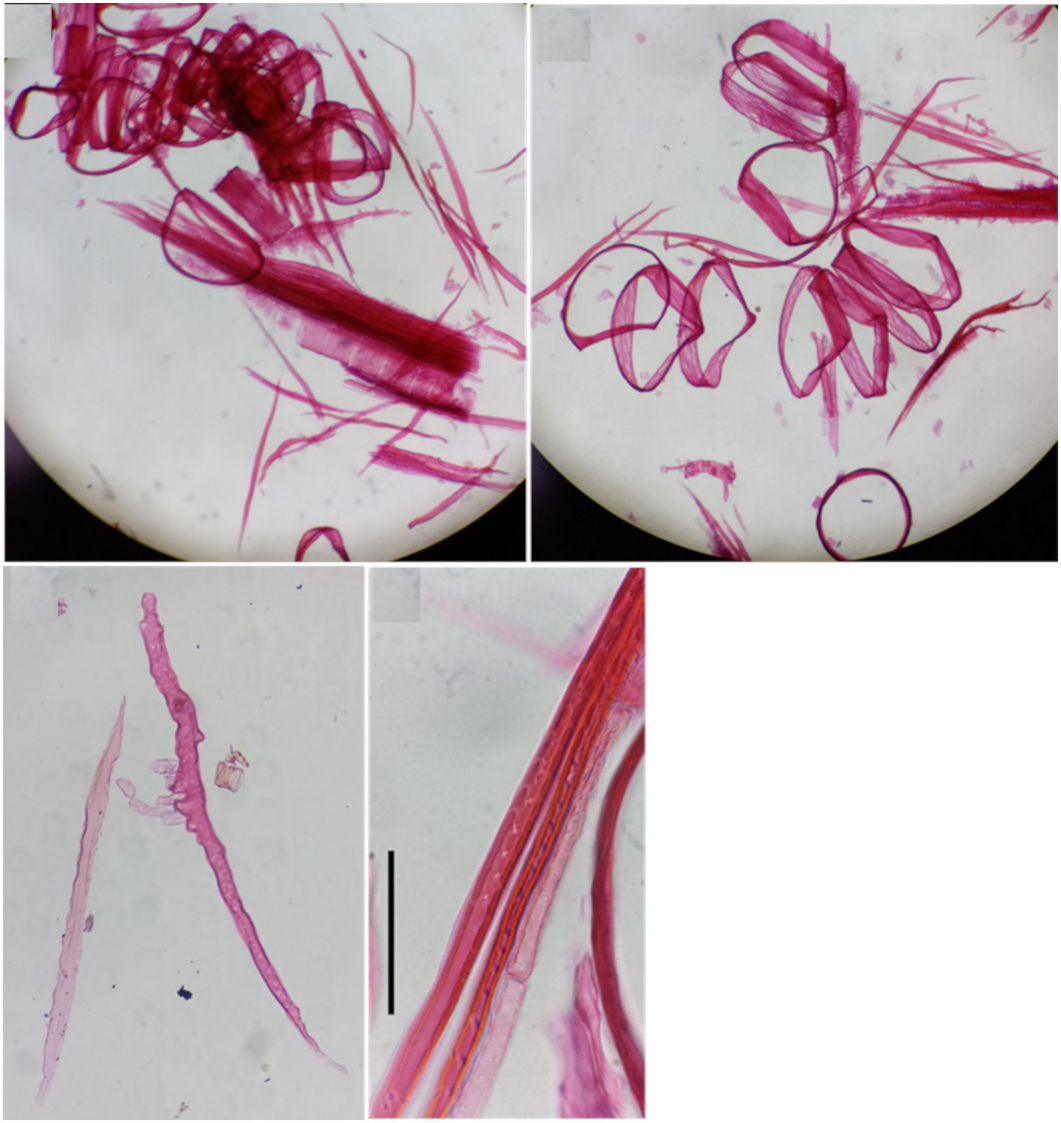
Anatomical features of angiosperm cells of *Merremia peltata* observed under the microscope after maceration using protocol P2. Scale bar = 100 µm.

P2 has been successfully used in a wide range of gymnosperms and angiosperms with extreme values of wood density. Particularly P2 has been successfully used in more than 50 species, belonging to at least 25 orders and 34 families (e.g., **Figures 6**–**9**).

**Figure 10:**
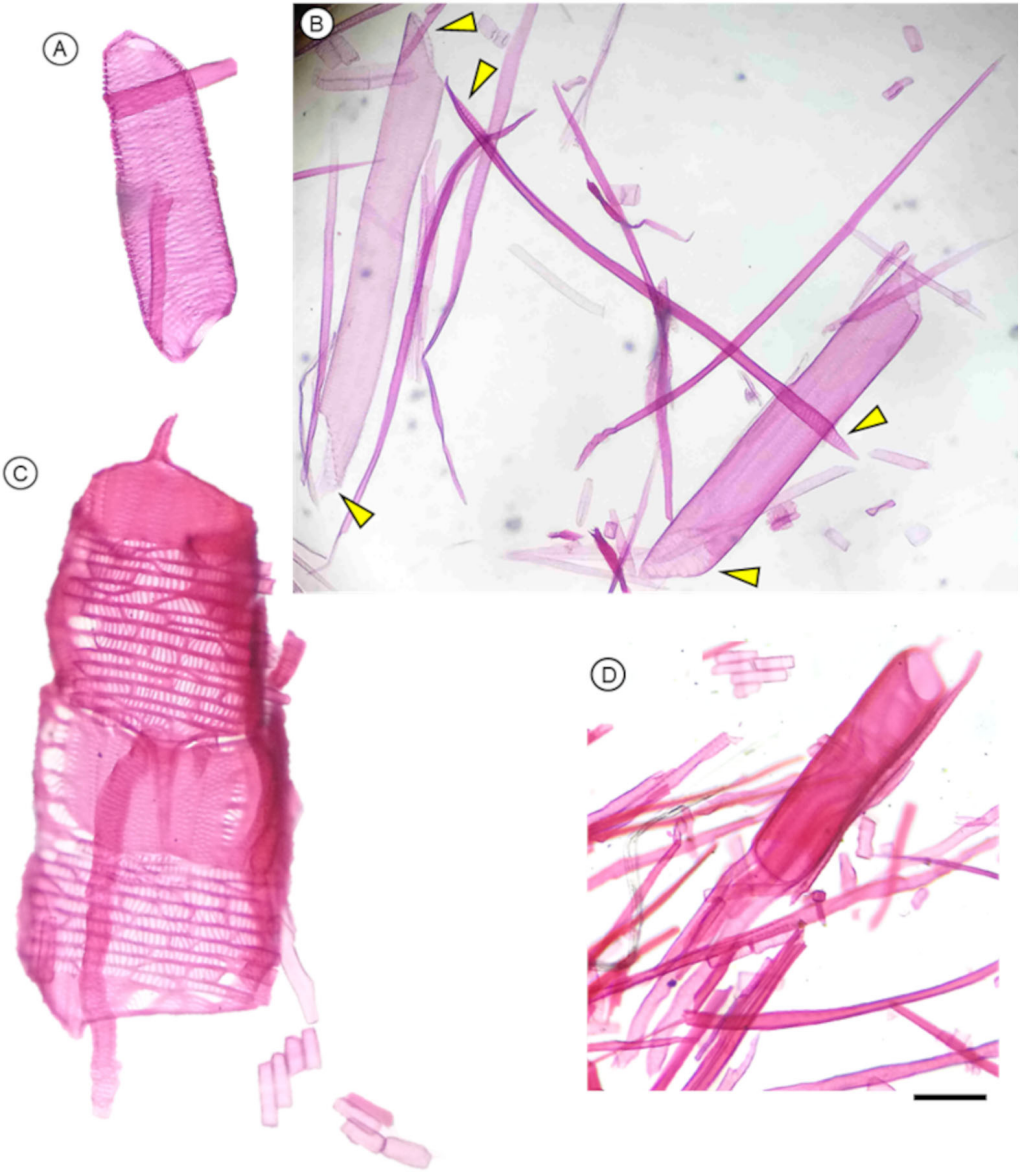
Anatomical characteristics of wood cells from low- and high-density angiosperms observed under the microscope after maceration following protocol P2. **(A)***Adansonia digitata* (Baobab) vessel element. **(B)***Austrobaileya scandens* vessel elements with scalariform plates (arrowheads) and fibers; note that one vessel element seems like a fiber due to its narrowness. **(C)***Quercus skinneri* earlywood vessel elements in connection forming part of a vessel, with vasicentric tracheids, and pits that connected radial parenchyma. **(D)***Quercus rubra* vessel element surrounded by vasicentric tracheids, fibers. Scale bar = 100 µm.

**Figure 11:**
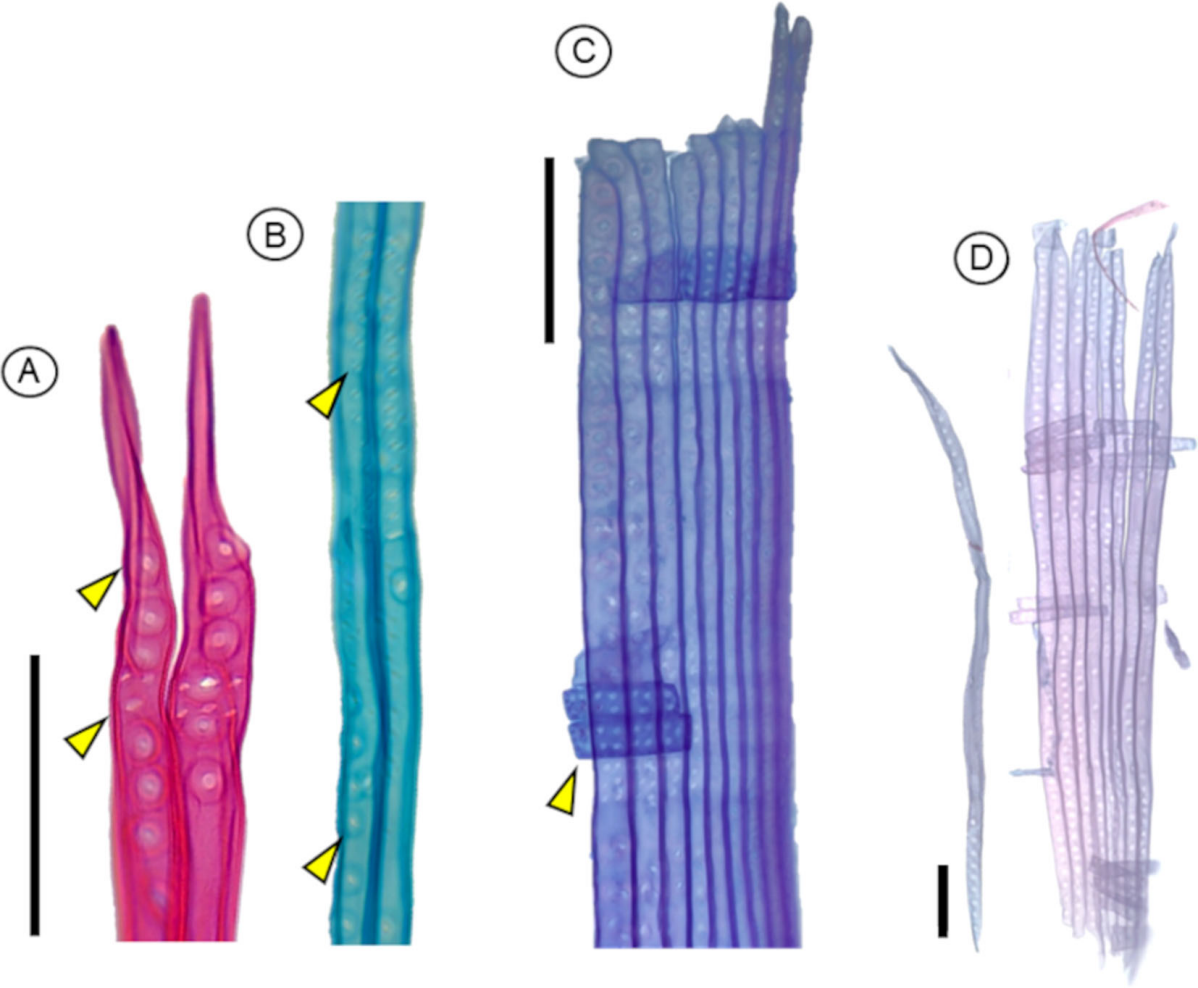
Anatomical characteristics of the tracheids of *Larix decidua* obtained using the maceration protocol P3. **(A)** Detail of pits in tracheid endings, and **(B)** in other tracheids between the endings, showing above pits that connected with radial parenchyma, and below with other tracheids. **(C)** Radial parenchyma (arrow) and tracheids with different diameters. **(D)** Group of tracheids of similar lenght with radial parenchyma, and a tracheid slightly shorter. Scale bars = 100 µm. Staining: Safranin + Astra Blue.

P3 has been proved to work on the following gymnosperm species: *Picea abies* (**Figures 10A, B**), *Picea glauca* (**Figures 7E**, **10C**), and *Larix decidua* (**Figures 10D**, **11**).

## 5 Discussion

While maceration is a valuable tool in anatomical research, it is essential to acknowledge that each method, including those outlined in this study, may present species-specific challenges. Based on our experience, we present the following observations and considerations to avoid common issues and improve outcomes.

*Observations and considerations*.

Maceration timing is an important factor to consider in cellular analysis, as too short maceration can leave cells undissociated. However, there is no single rule that applies universally, not even with Franklin’s solution. But the key advantage of Franklin’s solution is that in secondary xylem it does not appear to disintegrate cells after prolonged maceration, not even in species composed of parenchyma as a ground tissue (e.g., lacking imperforate tracheary elements, with vessels as the only lignified cell type). Low-density xylem tends to take more time than high-density xylem, but unlike the methods that use Jeffrey’s fluid, Franklin’s solution maintains the mechanical integrity of the samples, allowing more manipulation without significant risk of damage. However, for phloem, the thin-walled sieve elements and some non-lignified parenchyma cells present in the bark appear to be sensitive to damage by prolonged maceration with Franklin’s solution if the maceration is too long. Prolonged maceration of phloem can make the phloem sieve cells softer and more fragile, thereby increasing the risk of their damage or breaking during homogenization. Therefore, maceration of the phloem should be stopped as soon as the cells can be easily separated.

Contrary to common assumptions, xylem density does not consistently correlate positively with maceration difficulty or duration. In our experience, higher-density xylem often macerates faster, whereas certain low-density xylem, such as “bottle tree” types (e.g., baobab; **Figure 10A**), and low-density fern xylem, show much greater resistance to maceration (e.g., requiring days to weeks). These observations raise questions about the chemical factors underlying differential maceration rates.

Simply leaving the samples in the solution until they are at the required level of softness is all that is required, but it can help to budget for potentially longer maceration times for very low-density species. Protocol OP performs effectively on north-temperate conifers and angiosperms, where vessels, tracheids, fibers and fiber-tracheids, separate clearly within hours even under mild heating conditions (approx. 50 °C). Podocarps and Araucarians often result in maceration times of several days. With the exception of fern tracheids, herbaceous taxa seem to macerate efficiently.

As macerations involve observing single cells, it is essential for each cell to be stained as intensely as possible to observe its features readily. The best way to ensure this is to use strong staining solutions. For example, protocol P2 works well with a saturated solution of safranin, the stock has safranin sediment on the bottom) in 100% ethanol, and to avoid excessive rinsing that will de-stain the tissue. For protocol P2, just a quick rinse of the vial with 100% ethanol is necessary, followed by a quick dry with paper towels pushed into the vial to absorb excess ethanol and even drawing some of the residual staining solution out of the tissue. Then into clearing agent for 5 mins and discard (this first change is short because there is still ethanol that is de-staining the tissue; for example, a xylene rinse at this stage will tinge the solution pink because of the 100% ethanol with safranin entering into the xylene). Subsequent changes of clearing agent for approx. 10 minutes until the solution is completely clear usually eliminates all residues and perfectly conserves very intense staining.

Applying fluorochromes, such as Congo red, to stain macerated cells of xylem or phloem can be beneficial for detailed observation of cell structure with a confocal microscope. The provided protocols thus allow for creating 3D models of selected cells and quantitatively analyzing the ultrastructure of the macerated cells. For example, P1 can be applied to quantitatively analyze the structure of sieve plates in sieve tube elements by assessing the numbers and dimensions of sieve areas or sieve pores. This approach facilitates the quantitative analysis of the phloem cell ultrastructure compared to the traditionally used observations with SEM (Scanning Electron Microscopy; Knoblauch and Oparka, 2012; Truernit, 2014). Similarly, pit properties can be assessed for any of the cell types in xylem and phloem. However, for the quantitative analyses, it is important to note that maceration degrades some cell components, such as callose deposited in sieve pores. Similar structures cannot be observed in macerated cells and must be studied on selectively-stained sections instead.

### 5.1 Comparison between protocols P1, OP, P2 and P3

The protocol P1 has been optimized for universal use in macerating both xylem and phloem, whereas the remaining protocols have been tested for xylem only. Also, the maceration times vary significantly across the protocols. P1 is the fastest, taking only 2–6 hours, depending on the species and tissue type, while Protocols P2 and P3 require 24–48 hours or more of maceration, with P2 and P3 for even longer periods when no heating is applied (e.g., one month for *Picea* spp. and *Larix decidua*). Regarding protocol OP (P1 was optimized from this original protocol provided in **Supplementary Material**), the procedure is generally simpler and involves fewer steps, allowing the maceration of the samples, preparation of the slides, and their observation under a microscope within a single day. Due to the exposure of the samples to the higher temperature in OP, the maceration process is significantly faster compared to protocols P2 and P3, typically requiring only a few hours for most samples. OP is suitable for using glycerin as a mounting medium, whereas in P1 glycerin can also be used, but it might prevent adequate cell homogenization.

P2 ensures the appropriate maceration, staining and high-quality permanent slides in many different families, genera and species. Similar to protocol P3, P2 also allows us to observe the interconnection of xylem cells, which is useful for understanding the relationship between xylem structure and function.

The protocols largely differ in the technique and time of follow-up preparations of slides (summary of advantages and disadvantages of each protocol in **Supplementary Table S3**). P1 does not require any dehydration while Protocol P2 involves multiple ethanol dehydration steps which ensure clear observation and imaging of tissues. P3 allows for very rapid dehydration with minimal ethanol use, because the mounting medium does not require thorough sample dehydration.

Staining is generally a quick process in all our protocols, with P2 being the exception. However, as the primary goal of the maceration process is to obtain intact cells with intense staining, P2 has proven to be effective for achieving both. The types of stains used for staining in P1 are dependent on the examined tissue and considerably differ between those used for xylem and phloem as not all of the stains can be universally used to stain both tissues. Stains such as safranin or toluidine blue, which are frequently used for staining xylem members, typically do not stain non-lignified phloem sieve tube elements, or the staining is very inconsistent. In contrast, Astra blue and Congo red can be applied to both tissues with good staining results. To study phloem ultrastructure in particular, we recommend using Congo red, despite its considerable toxicity, as it can be observed both in light and fluorescence. However, for basic cell type identification and quantitative evaluation of cell dimensions, the usage of Astra blue is sufficient.

P3 uses minimal volumes of ethanol for dehydration, as the mounting medium work well (no cloudiness) with up to around 20% of water content. To obtain the results using P3 no clearing agent was used; however, its use is mentioned as an optional step if needed. In P3, the combination of Astra blue and safranin is used, and apparently, this might help if safranin destains (due to its water solubility), as Astra blue remains.

The mounting steps are relatively short in all protocols, typically taking between 5 to 10 minutes to apply the mounting medium and cover slip. However, P3 requires considerably longer time for solidification of the mounting resin in an oven at 65 °C from 3 days, and up to 3 months to fully harden.

### 5.2 Advantages and disadvantages of producing non-permanent slides and permanent slides

Given the time and effort required to collect and macerate samples, we highly recommend preparing permanent slides (protocols P2 and P3 provide examples of mounting technique), or storing the material in a freezer or a fixative for possible later use (e.g., protocol P1).

Depositing permanent slides in collections, rather than discarding them), is an excellent practice, as they can be useful indefinitely for future studies at minimal additional cost. The preparation of permanent and semi-permanent slides can be useful for capturing images at any time later, keeping them as reference material, or conducting further measurements, observation or analysis later on. However, there are disadvantages to permanent slides. For example, certain mounting resins (e.g. the one used in P3) can last up to 3 months in an oven to fully polymerize inside an oven, and, without heating (at room temperature) more than two years. Another disadvantage of certain mounting resins is that they might shrink with heat (e.g. the one used in P2, so, no heating is applied). However, when fully solid, a mounting resin protects tissue from deterioration due to humidity, fungi or bacterial action, and other factors, and slides can be stored easily (e.g. inside slide boxes).

Furthermore, when preparing permanent slides, inadequate dehydration results in cloudy preparation that often, in addition to being difficult to observe under the microscope because of the abundance of water droplets, often de-stain over time. However, inadequately dehydrated macerations, or those with large air bubbles or old mounting resin that has become opaque, can be restored by soaking off the coverslip in a clearing agent, carefully removing the old coverslip and collecting any cells stuck to it, and re-mounting. Another possibility that reduces the risk of cloudiness, is to use a clearing agent and a mounting medium that work efficiently even with a small percentage of water content (as the one recommended as optional in P3).

In contrast to permanent slides, preparation of non-permanent slides is much faster, easier, less laborious and requires less chemicals. P1 offers storage of macerated and stained cells in Eppendorf tubes for one or two weeks without a significant negative impact on the macerated cells. Similarly, making semi-permanent slides by sealing the sample with nail polish allows for storing the samples for a relatively long time to be used for additional examination under a microscope, if needed. Preparation of slides *de novo* from stored samples, though it is more laborious, can be made within a few hours.

Glycerin as a mounting medium to produce semi-permanent slides (as in protocols OP in **Supplementary Material**, and P1) offers a balance between cost, time, and efficiency. Several advantageous properties include a lower evaporation rate than water, a high refractive index, being inexpensive, and being non-toxic. However, it also presents important drawbacks: glycerin can dissolve some dyes from the stained cells, such as Safranin or Congo red, which may decrease the contrast of the cells in time. Moreover, glycerin also may run off a tilted slide, and even when the preparation is quickly sealed (lute), it might eventually evaporate. Therefore, it is always useful to seal the cover slip with nail polish when using glycerin. Furthermore, while imaging using a 100× oil immersion objective, the slight pressure exerted by the lens through the oil can displace cells on the slide, potentially affecting their positioning during observation. However, there are water-soluble mounting mediums and techniques that can help stabilize the cells in the slides for imaging (e.g., Zander, 2014).

## 6 Conclusions

Maceration is already a widely used technique in various fields of research, such as anatomical and ecological studies. However, in most laboratories, maceration methods often evolve through tradition or trial-and-error. These adjustments are often made without being documented, which limits their reproducibility and hinders method standardization. To address this gap, we presented three updated and clearly defined maceration protocols. These have been designed to improve the overall efficiency of the maceration process, reduce safety risks, and enhance the quality of individual-cell analysis. The protocols are adaptable to a wide range of woody species, including both gymnosperms and angiosperms, and offer flexibility to accommodate different research goals and species-specific requirements. As we recognized the limitations that may exist in some research environments, we provided practical alternatives for settings lacking certain facilities, such as fume hoods. This makes the protocols more accessible and applicable across diverse laboratory conditions.

We provided a detailed description of different protocols and considerations helping researchers choose the most suitable protocol based on their available time and specific experimental needs. Each protocol has its advantages and disadvantages, but the steps can be mixed and combined to obtain the best results in the shortest time with the available materials.

All the protocols presented here are primarily designed for the maceration of the xylem, but the P1 has been proven to work well with phloem of many species and allows observation of delicate sieve tube elements and sieve cells in detail. Providing these protocols might help to streamline the research in xylem and phloem anatomy and to encourage continued investigation of the structure-function relationships in both vascular tissues and plant responses to environmental conditions. We consider that there are broader implications of maceration in ecological, evolutionary, and industrial contexts, where its applications extend beyond pure anatomical research to areas such as forestry, conservation, and biotechnology.

The updated protocols presented here provide a versatile toolkit for scientists exploring the intricate relationships between anatomy and environmental factors, ultimately advancing our understanding of plant biology and forest ecosystems. By presenting these maceration protocols, we expect to contribute to unlocking the full potential of this technique for ecological, evolutionary, and industrial studies.

## Data Availability

The original contributions presented in the study are included in the article/**Supplementary Material**. Further inquiries can be directed to the corresponding author.
