# Supplementary material for: Providing maceration protocols for xylem and phloem research
Source: Front Plant Sci. 2026 Jan 22;16:1740174. doi: 10.3389/fpls.2025.1740174 (PMC12872850; doi:10.3389/fpls.2025.1740174)
Supplement: Supplementary file 1 [file DataSheet1.pdf]

frontiers

Supplementary Material

Specific items, reagents used or suggested for each protocol (P)

P1. Ultrasonic homogenizer (e.g., UP200S, Hielscher Ultrasonics, Germany). Digital microscopic camera e.g., Levenhuk DTX TV, Levenhuk, USA. Kimwipes. The folder mentioned for storing the slides can be found with names such as: envelope microscope slides made of cardboard, cardboard case for microscope slides.

Staining of macerated cells:

- Recommended stains for xylem maceration include: i) Safranin dissolved in water or 50% ethanol: stains lignified cell walls into pink or red; ii) Fast green or Astra blue dissolved in water: color cellulosic primary cell walls into green or blue color, therefore highlighting less lignified tissues and non-lignified ones (although in maceration may not provide consistent results); iii) Toluidine blue dissolved in water: stains both lignified and cellulosic cell walls into green to blue color; iv) Congo red: stains cellulosic cell walls into red color.
- Recommended stains for phloem maceration include: i) Astra blue: stains cellulosic primary cell walls into blue color, ii) Congo red dissolved in water: stains cellulosic cell walls red.
- Using contrast-enhancing dyes is useful for highlighting different cellular components and facilitating detailed analysis of cell structure. However, maceration degrades some cell components in phloem (e.g., callose in sieve tube members and sieve cells) and alters the cell wall properties. Therefore, several stains commonly used to stain sections may not stain macerated phloem cells, or their use may not provide consistent results.

P2. Entellan as mounting resin, xylene as clearing agent**.** Be aware of the jar or container composition used while heating. For example, polypropylene (PP) can withstand temperatures up to 120 °C. A classical polyethylene terephthalate (PET) starts deform around 70°C.

P3. Euparal as mounting resin. The use of a clearing agent in this protocol is optional. If needed, ROTI Histol can be used as a clearing agent. For imaging: a trinocular optic microscope (e.g., OLYMPUS CX43) with a digital camera (e.g., PROMICAM PRO HDMI, 16 Megapixels with a native software included; or a third-party software can be used, such as ToupView), and ideally a mount adapter disc (e.g., U-TV0.5XC-3-7 C) that allows a wider field of view. For calibrating each objective of the microscope, a stage micrometer was used (e.g., levenhuk, 1DIV = 0.01). For stitching and aligning pictures, a software can be used (e.g, PTGui, Image J, PICOLAY).

Bio Clear as a clearing agent can be a safe alternative to xylene, and it can be used with both Euparal and Eukitt mounting resins.

Protocol OP for maceration and mounting in non-permanent and semi-permanent slides

Steps (1-15; Supplementary Figure S6-S10) are the following:

1. Prepare a solution consisting of one part of fresh hydrogen peroxide (35% H_2O_2), one part of glacial (absolute) acetic acid, and one part of water in a glass flask.
2. Cut the wood block into small pieces (1 – 2 mm thick) using a razor blade.
3. With tweezers, put the cut pieces of samples into the small glass vial (20 mL) or into the small Erlenmeyer flask (25 – 50 mL) and add approx. 5 - 10 mL of the maceration solution.
4. Close the flask with a small funnel or aluminum foil to minimize the loss of maceration solution during sample heating by evaporation. Avoid closing the flask tightly with a cup to minimize the risk of explosion during heating the sample. Do not recycle the old maceration solution, as the hydrogen peroxide degrades during boiling.
5. Place the flask on a hot plate and heat the samples for 2 - 6 hours, depending on the species and sample type. Maintain the temperature of the solution between 70-90°C (avoid ebullition).
6. We recommend proper testing of the duration of maceration with respect to the sample identity and examined cell type. Maceration for 2 – 3 hours and 3 - 4 hours is sufficient for separating individual phloem and xylem cells in various woody species, respectively.
7. When the maceration is done, the samples should be thoroughly washed in water to stop the maceration. Remove the flask from the hot plate with tongs and let it cool down for 10 min.
8. Pour the solution with macerated samples into a small beaker. Subsequently, transfer the samples into another beaker filled with distilled water using tweezers. We recommend placing samples into a cell strainer floating in water. Wash the samples repeatedly by submerging the strainer with samples in fresh water until they no longer smell acidic.
9. Use a brush pencil or tweezers to place a macerated sample into a drop of water on a slide
10. Gently separate the macerated sample into smaller pieces using two dissection needles
11. Add more water with a pipette to the sample and cover the samples with a rectangle coverslip. Repeatedly raise and lower the coverslip to tease the sample between the slide and the coverslip.
12. Remove the excess water from the slide using a piece of filter paper. Avoid removing too many macerated cells. A pipette can be used instead of the filter paper, but it may remove a large number of macerated cells from the slide.
13. Stain the macerated samples by adding several drops of a 0.1% - 0.5 % (w/v; weight/volume) aqueous solution of Toluidine blue (*i.e.*, 0.1 – 0.5 g) dissolved in 100 mL of distilled water. Tease the samples by using the coverslip to facilitate penetration of the stain to all cells in the sample. Alternatively, Safranin can be used as a substitute for Toluidine blue as a staining agent.
14. After 3-4 minutes of staining, replace the stain thoroughly with fresh distilled water several times until the solution is clear. Use piece of filter paper to avoid removing excessive amounts of macerated cells from the slide.
15. Remove the redundant water and close the coverslip. Slowly lay down the coverslip using the needle to squeeze out all the bubble from one side to the other to avoid bubble formation.

- For the preparation of non-permanent slides, a drop of water or glycerin (50% - 90 %) is placed on the slide with the macerated cells. We do not recommend using safranin as glycerin dissolves it and decreases the contrast of the cells. A cover slip is carefully applied, and excess water/glycerin around the edges is wiped off with a piece of filter paper. When water is used as the mounting

medium, placing the slides in a box with wet paper towels helps prevent evaporation, allowing the slides to be preserved longer without the need for water refilling. Alternatively, nail polish can then be applied to seal the edges of the cover slip to prevent water or glycerin leakage. These semi-permanent slides must be stored flat in a folder to avoid the risk of leakage (e.g., do not place upright in a slide box). Once prepared, the slides containing water as a mounting medium remain stable for a few weeks when stored in the fridge, while slides containing glycerin can be stored for several years.

OP. Glycerol (the scientific name for the pure compound, typically used in laboratory and pharmaceutical contexts) and glycerin (the commercial term, contains at least 95% glycerol along with water and other impurities) refer to the same chemical compound (propane-1,2,3-triol, $C_3H_8O_3$).

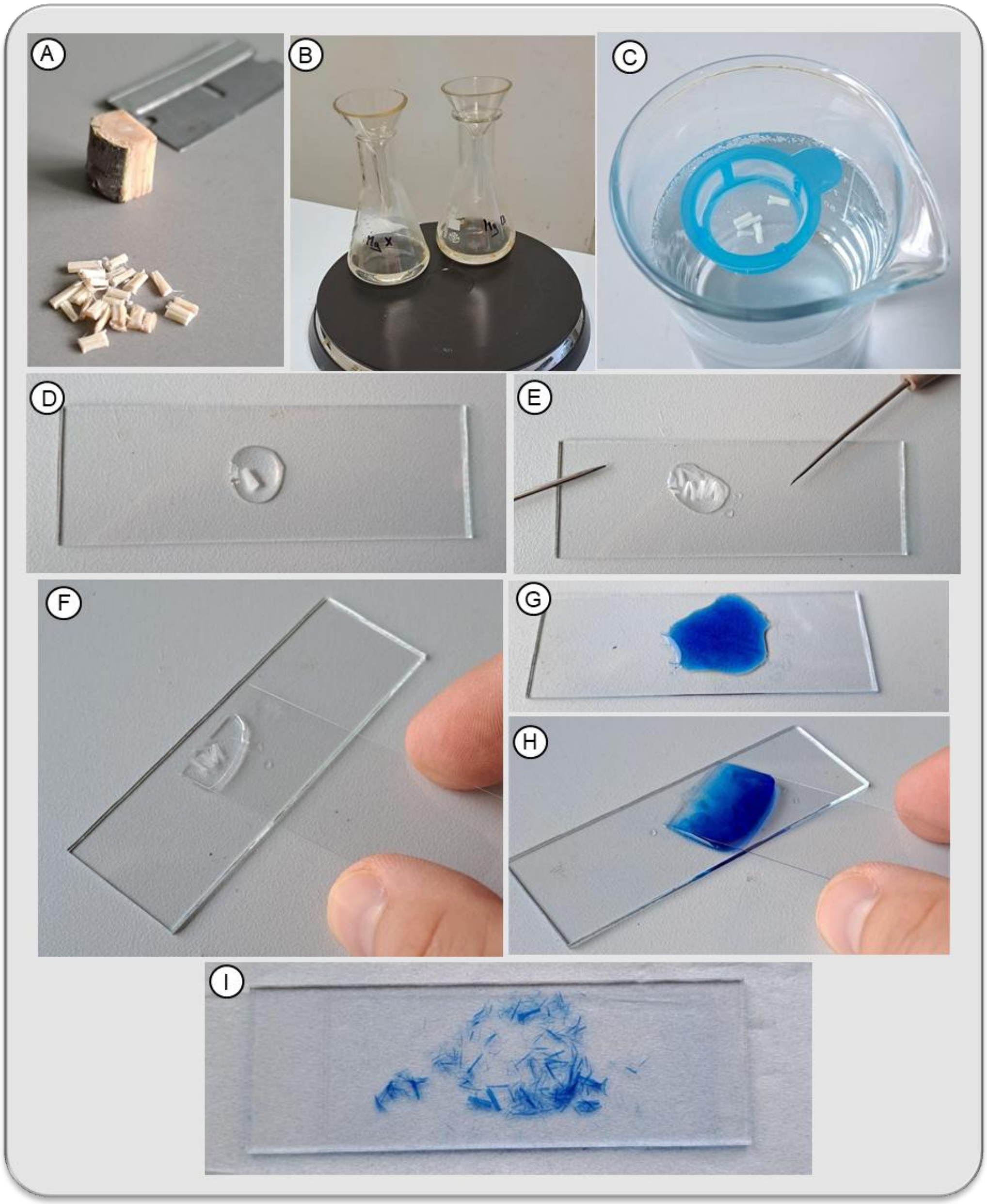

Supplementary Figure S1. Photos showing a summary of the procedure of Protocol OP. (A) Cutting wood sample into 1-2 mm thick pieces using a razor blade, (B) putting wood pieces into small Erlenmeyer flasks filled with maceration solution and heating them to 70-90°C for 2-6 hours, (C) washing the pieces of wood in water using a cell strainer, (D) putting washed samples on a slide into water, (E) separating piece of wood into smaller pieces using two needles, (F) teasing the sample between the coverslip and slide to separate individual cells, (G) adding few droplets of Toluidine blue to stain the macerated cells, (H) raising coverslip to facilitate dye penetration and staining of the cells , (I) final non-permanent slide.

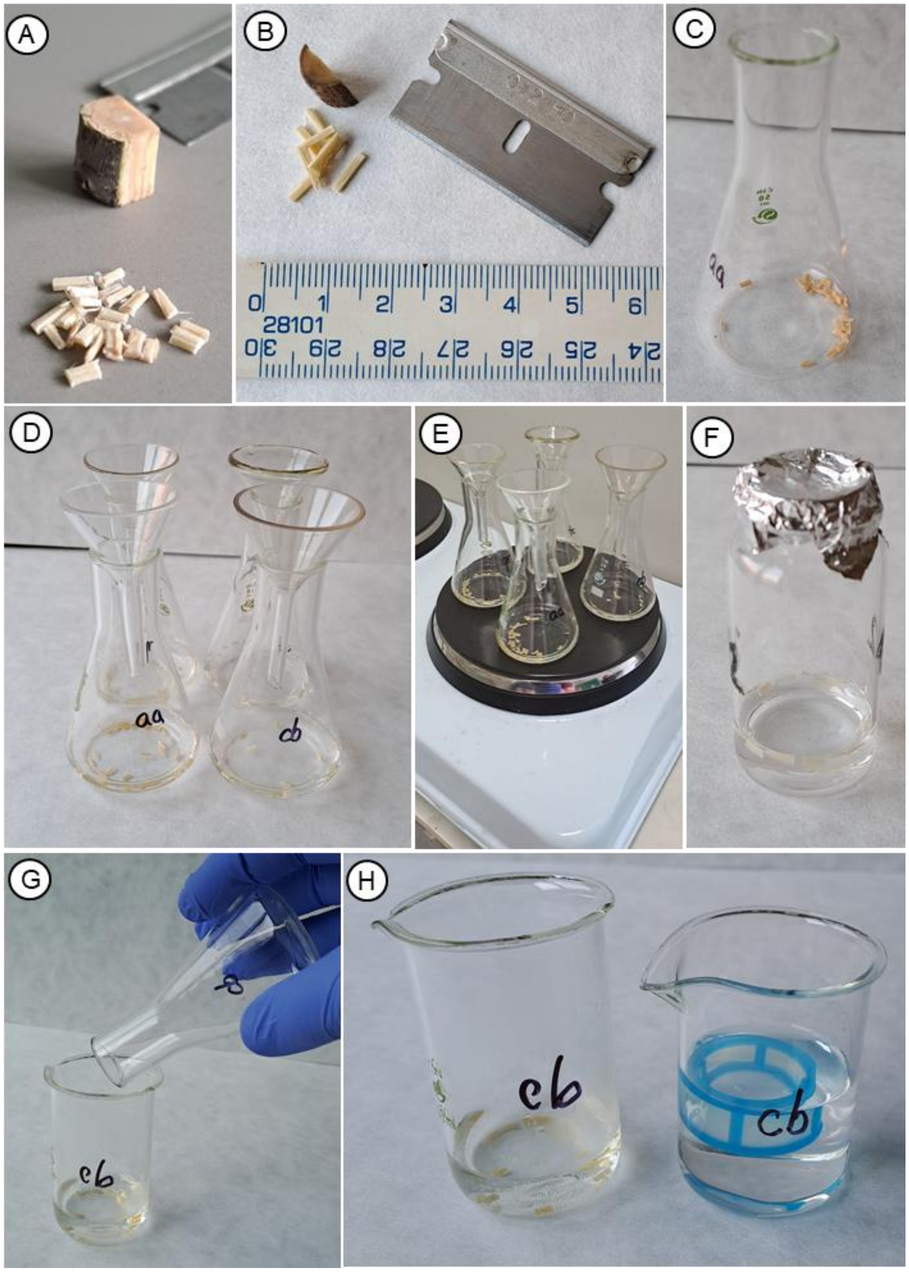

Supplementary Figure S2. Photos showing ideas for maceration steps.

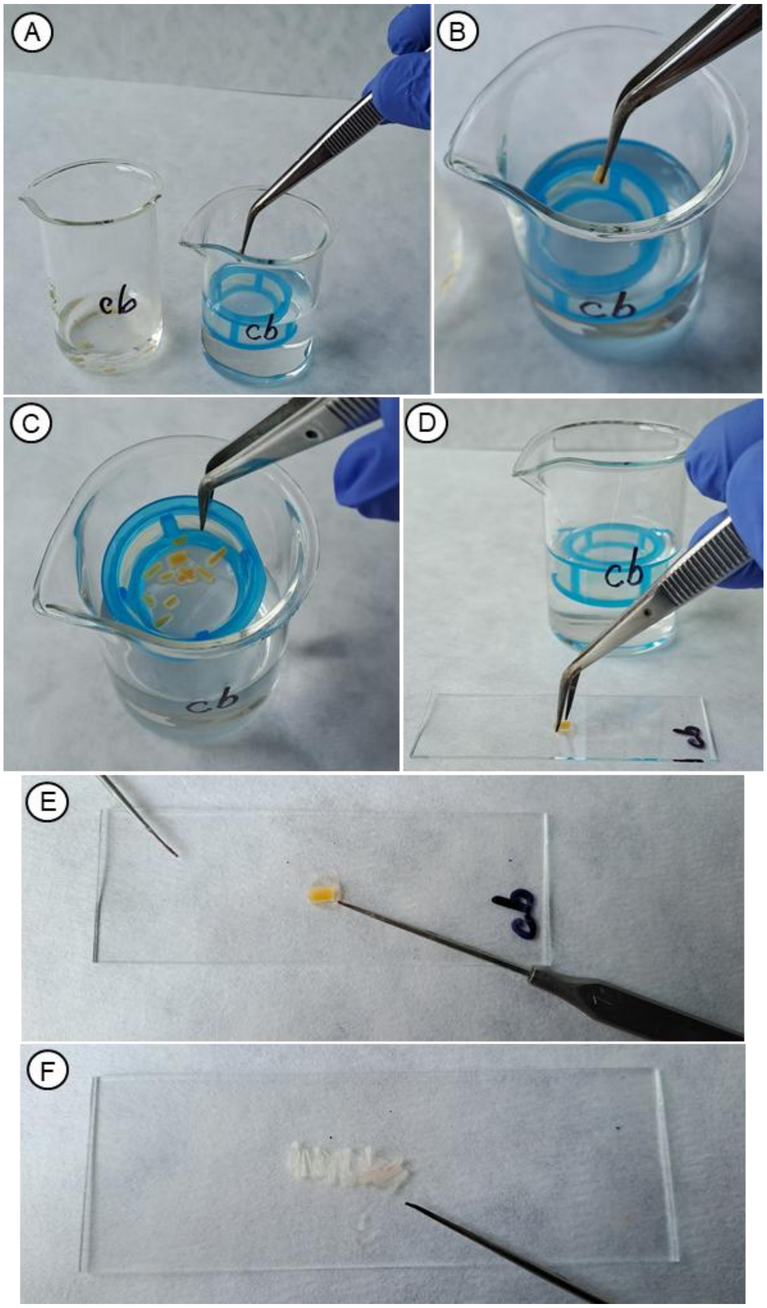

Supplementary Figure S3. Photos showing ideas for mounting steps for non-permanent slides.

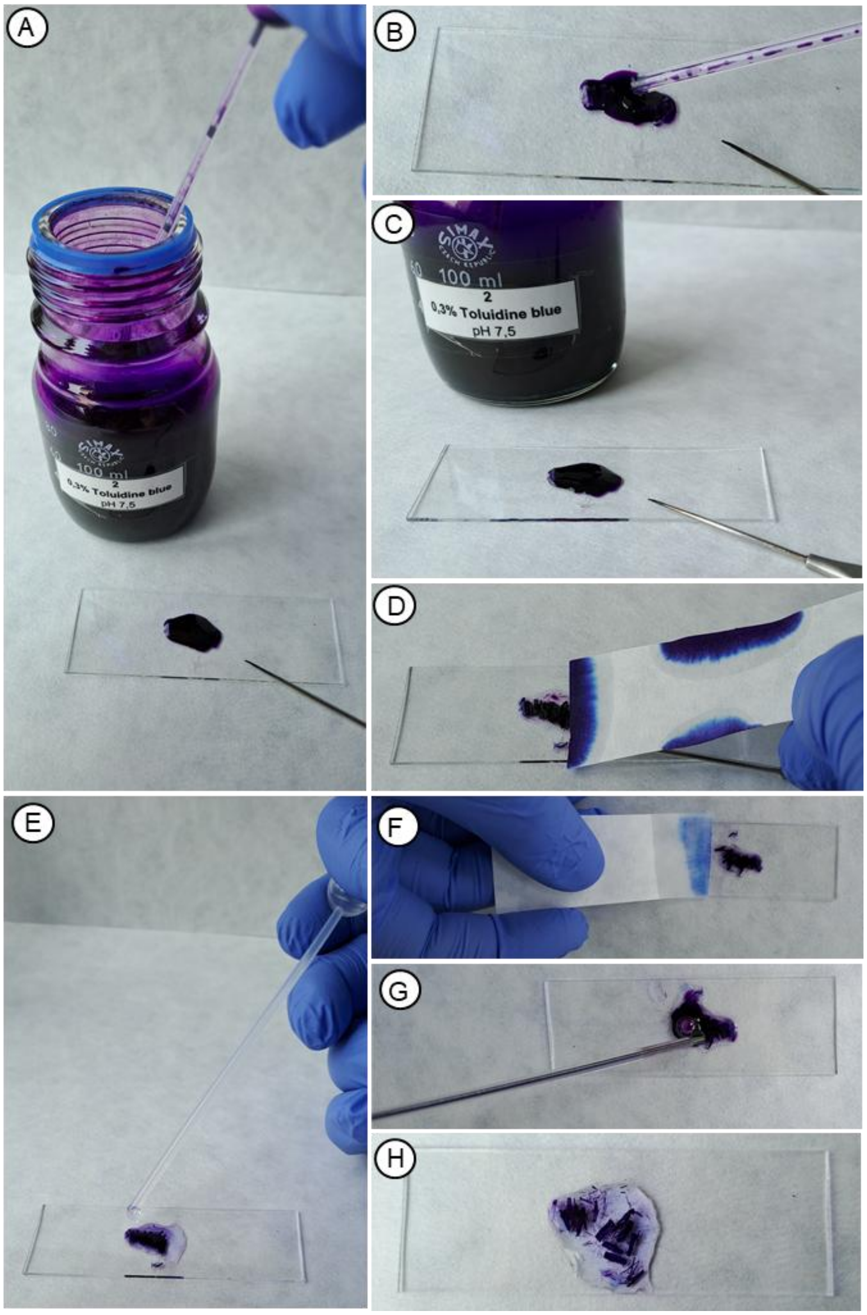

Supplementary Figure S4. Photos showing ideas for staining steps.

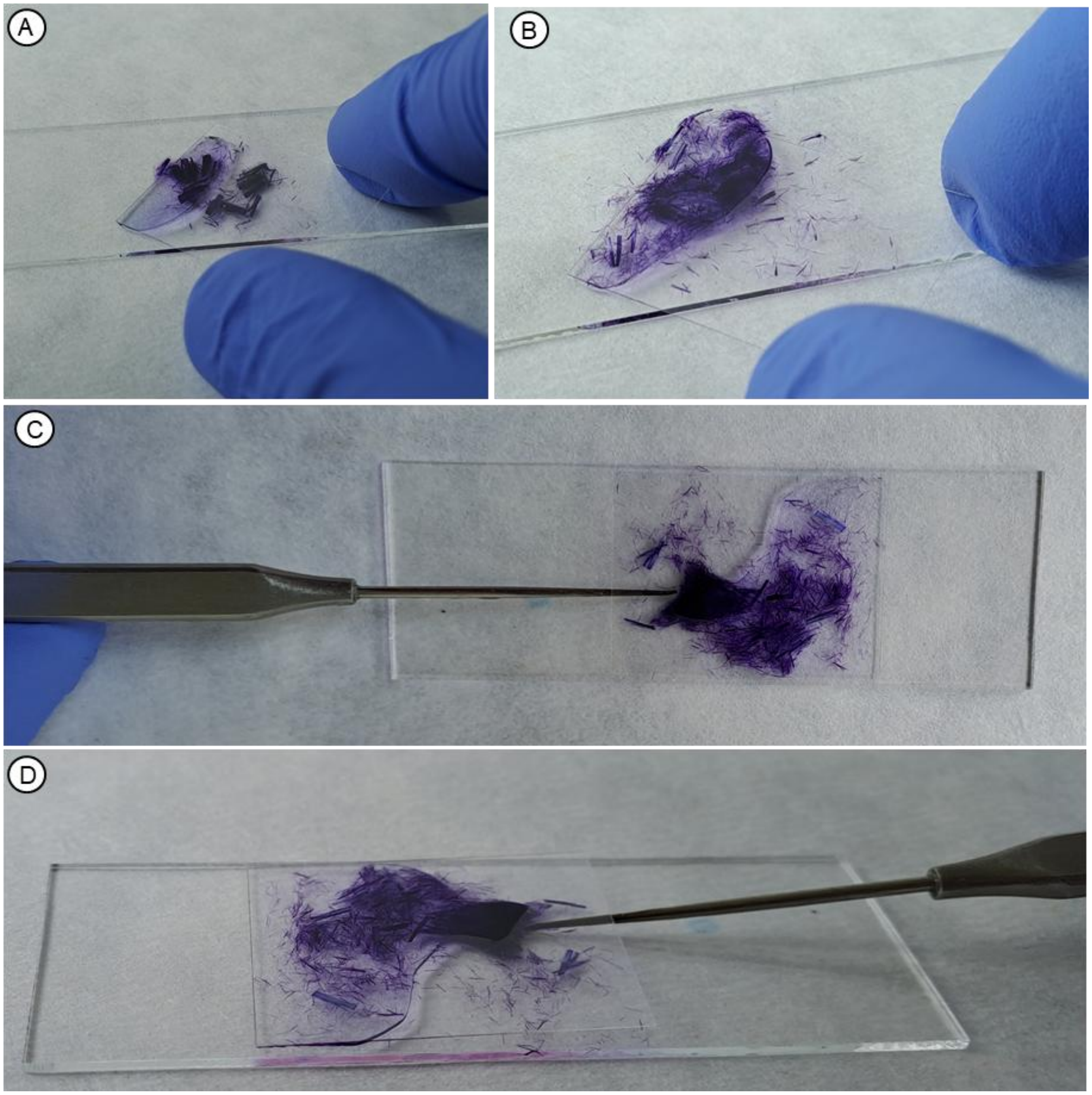

Supplementary Figure S5. Photos showing ideas for staining steps.

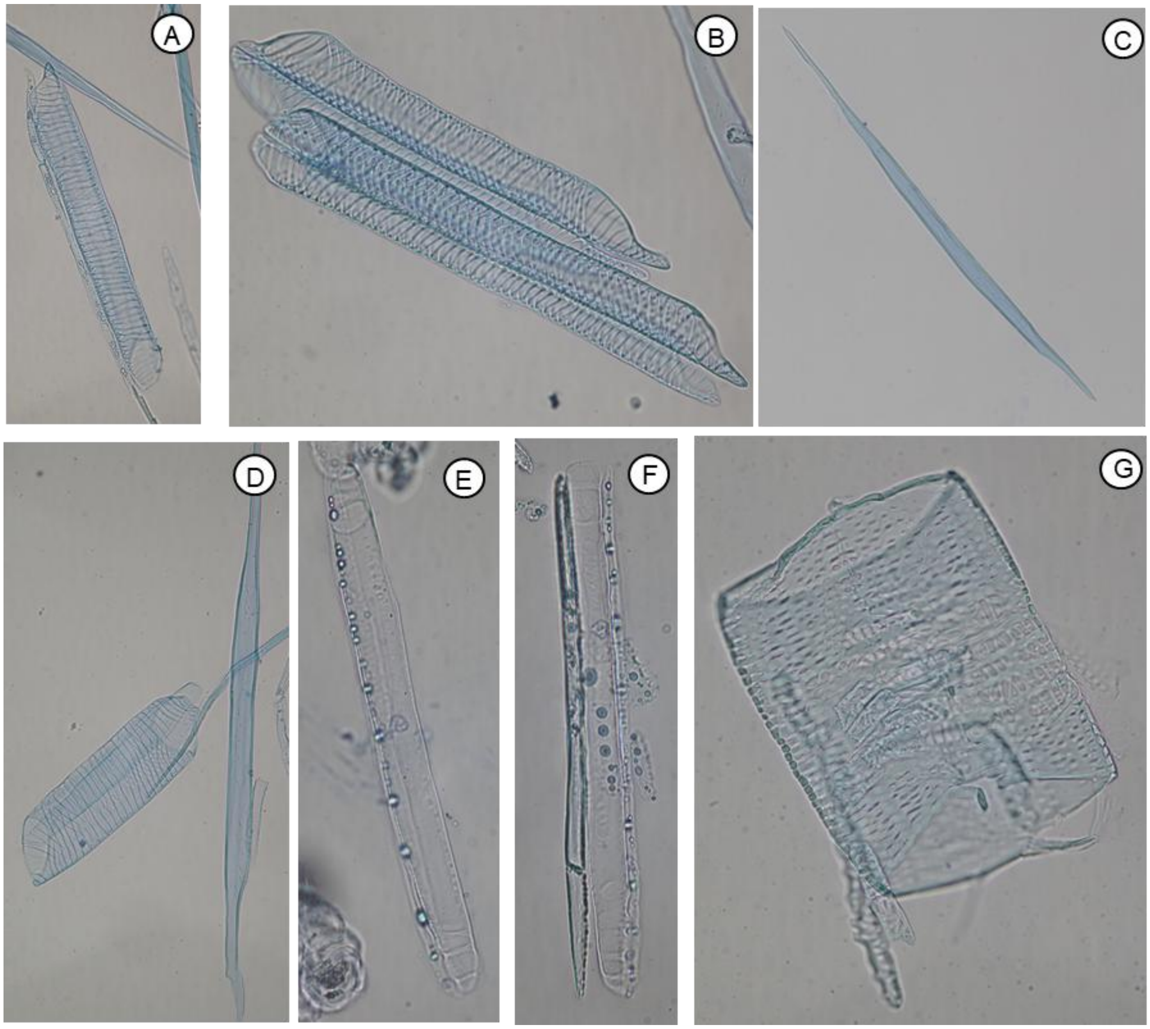

Supplementary Figure S6. Examples of macerated cells prepared with the Protocol OP. (A) *Tilia cordata*, vessel member with attached axial parenchyma cells. (B) *Tilia cordata*, a group of vessel members. (C) *Tilia cordata*, libriform fiber. (D) *Tilia cordata*, a vessel member and a libriform fiber. (E) *Tilia cordata,* a sieve element. (F) *Tilia cordata,* a sieve element with attached companion cells. (G) *Quercus robur*, earlywood vessel member.

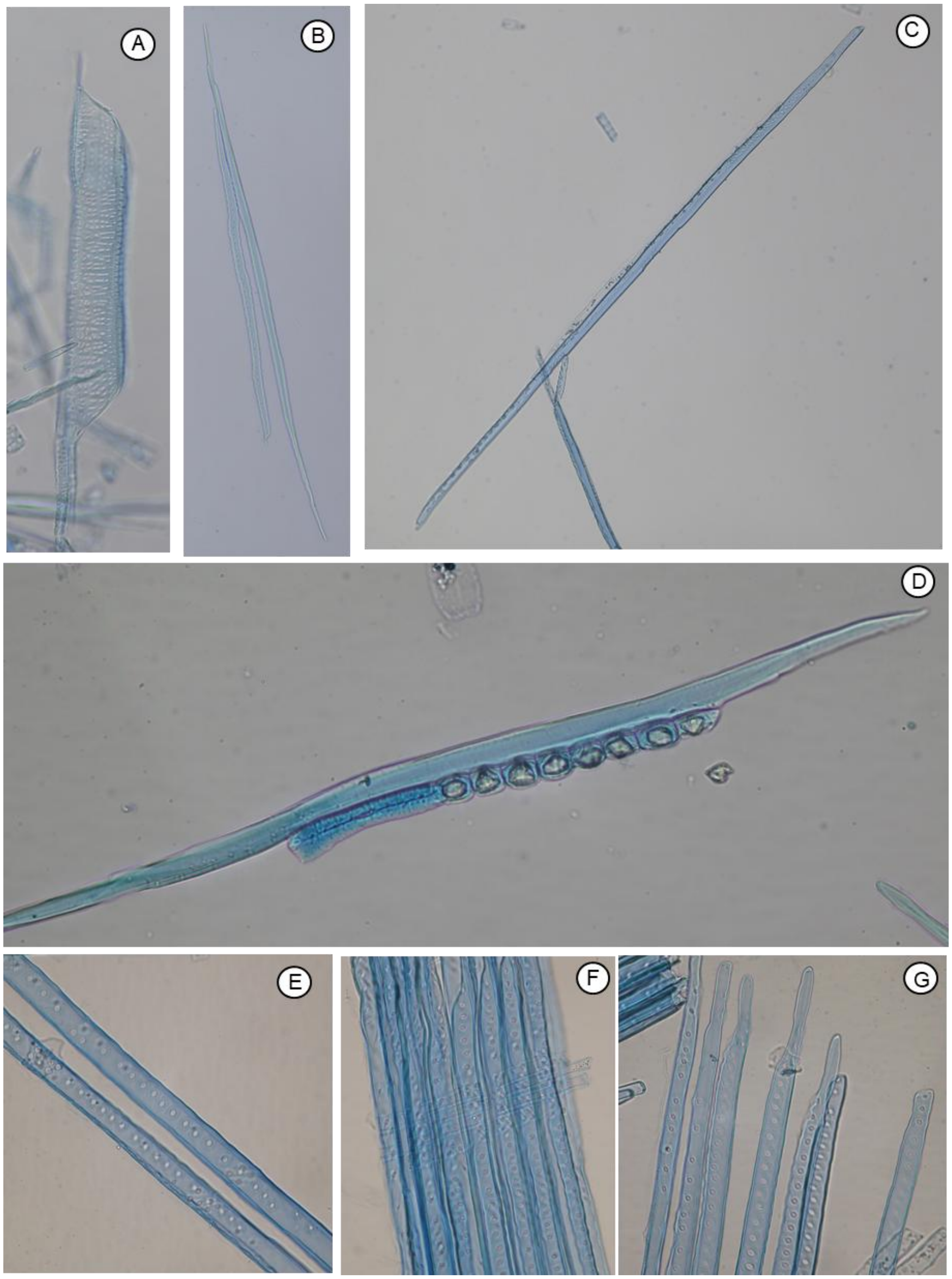

Supplementary Figure S7. Examples of macerated cells prepared with the Protocol OP - earlywood vessel member. *Quercus robur*. (B) libriform fiber and fiber tracheid. *Quercus robur*. (C) a tracheid with attached axial parenchyma. *Metasequoia glyptostroboides*.
(D) *Quercus robur*, bark fiber with attached sclereid and some parenchyma cells. (E) *Metasequoia glyptostroboides*, detail of tracheid surface with bordered pits. (F) *Metasequoia glyptostroboides*, several tracheids with a remnant of a ray. (G) *Metasequoia glyptostroboides*, ends of multiple tracheids.

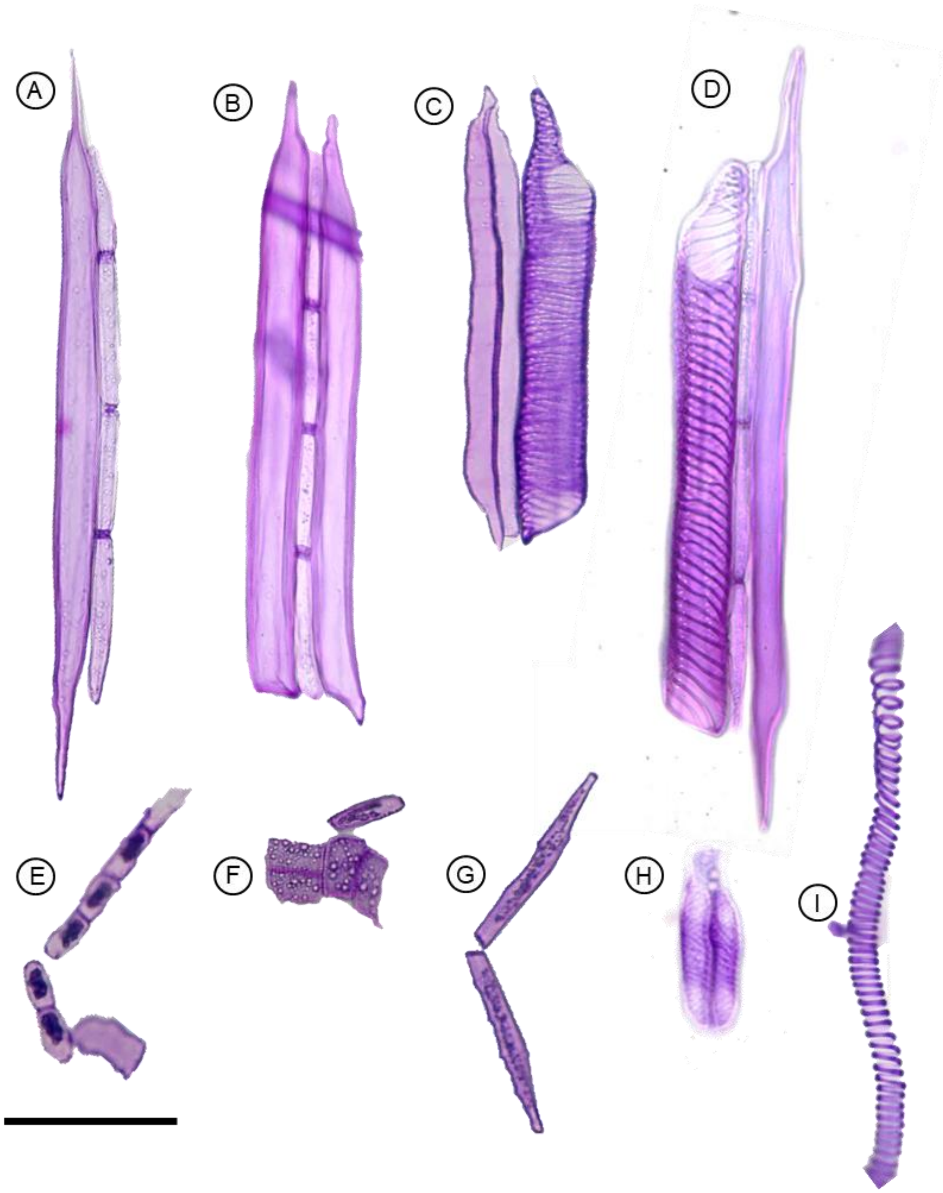

Supplementary Figure S8. Photographs from *Tilia cordata* xylem macerations coming from Protocol OP; Toluidine blue 0.3% was used as staining. (A) and (B) libriform fibers with axial parenchyma cells. (C) vessel element with helical thickenings and libriform fibers. (D) vessel element with helical thickenings and attached axial parenchyma cells and a libriform fiber. (E) radial parenchyma cells with nucleus. (F) radial parenchyma cell with contents (probably starch). (G) axial parenchyma cells. H- vessel elements. (I) helical thickenings of the primary xylem. Scale bar = 100 µm.

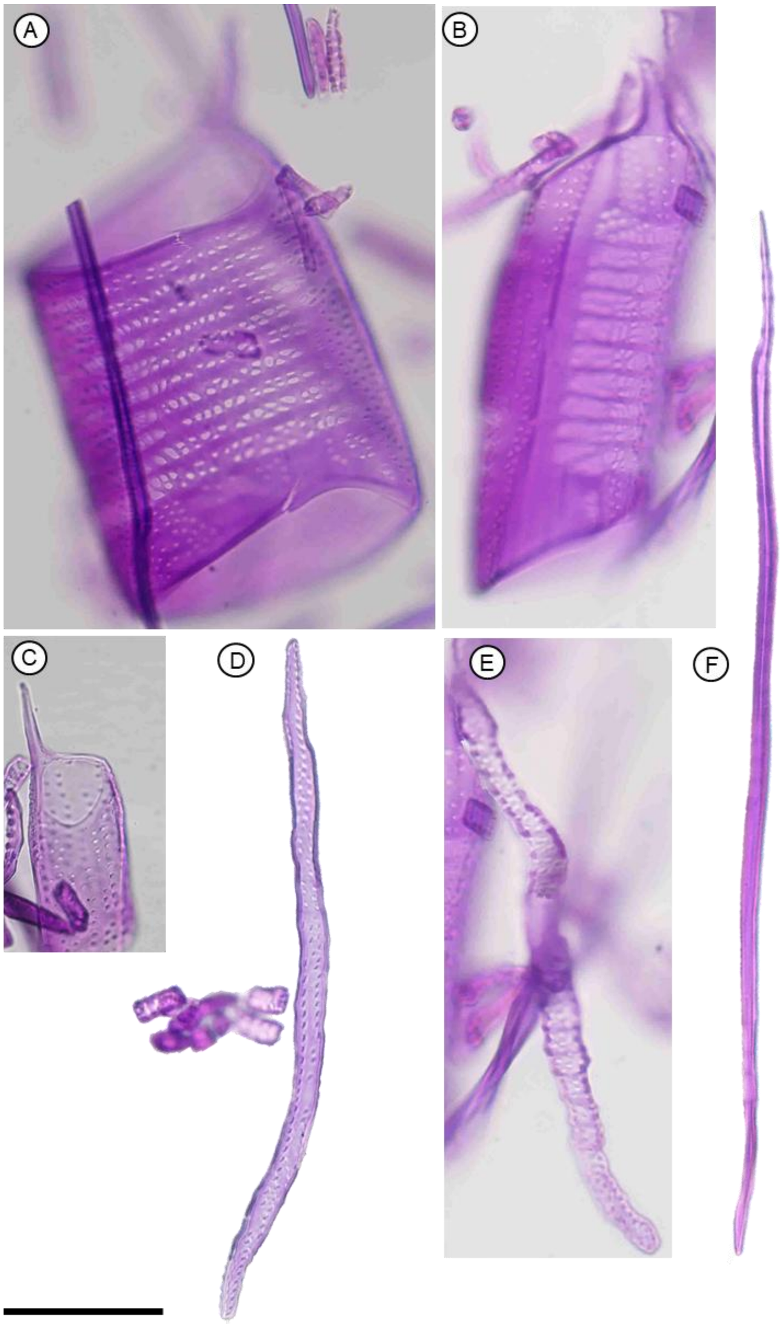

Supplementary Figure S9. Photographs from *Quercus rubra* xylem macerations coming from Protocol OP; Toluidine blue 0.3% was used as staining. (A) and (B) earlywood vessel elements showing vessel-ray pitting. (C) latewood vessel element. (D) radial parenchyma cells and fibrotracheid. (E) vasicentric tracheid with irregular shape and distinctly bordered pits in radial and tangential walls. (F) libriform fiber. Scale bar = 100 µm.

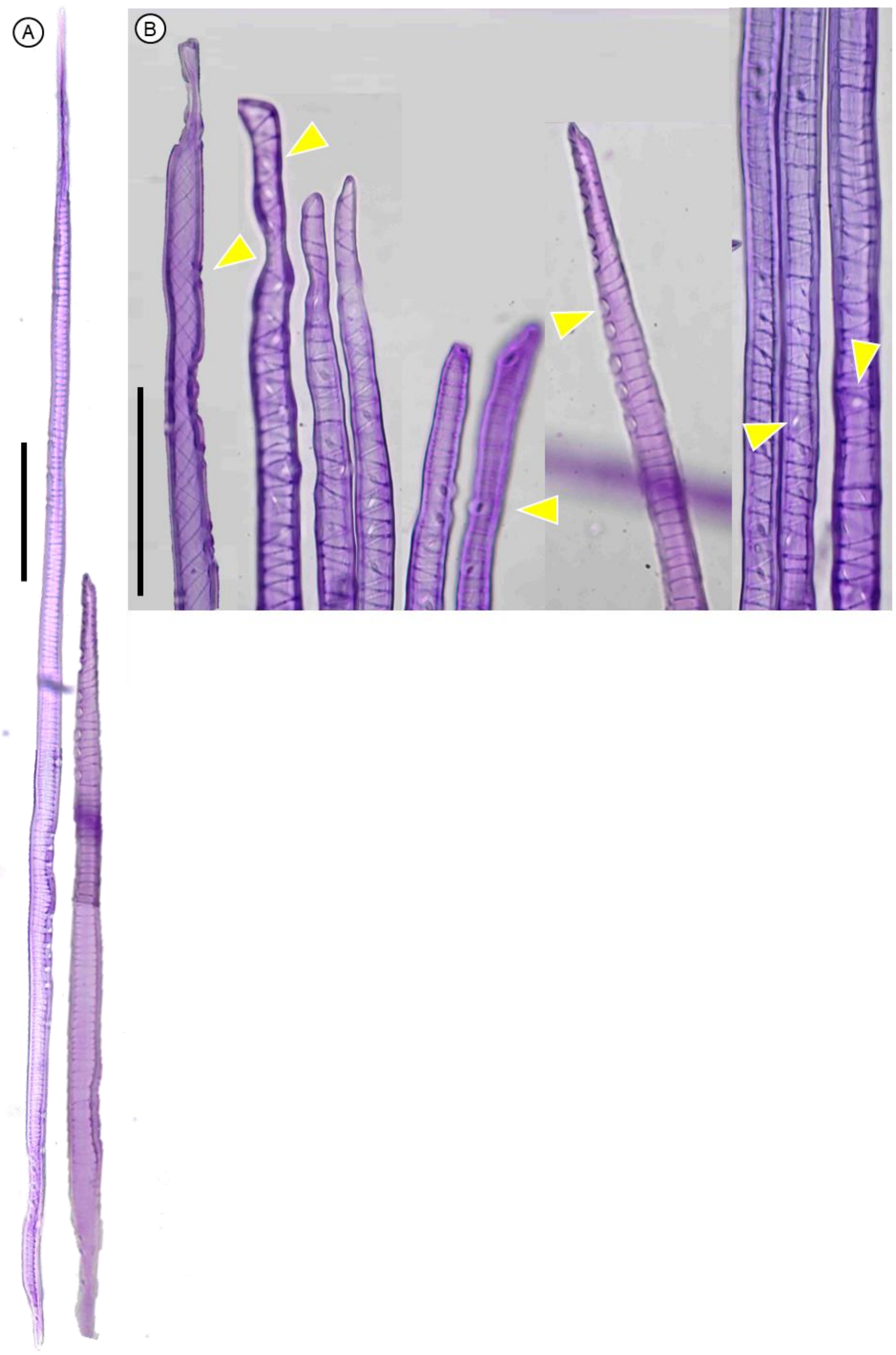

Supplementary Figure S10. Photographs from *Pseudotsuga menziesii* xylem macerations from protocol OP, showing tracheids; Toluidine blue 0.3% was used as staining. (A) Complete tracheids of different sizes in the same sample. (B) Details of bordered pits (arrows) and helical thickenings (single and narrowed spaced) in the inner face of tracheids. Scale bars = 100 µm.
