# Supplementary material for: Providing maceration protocols for xylem and phloem research
Source: Front Plant Sci. 2026 Jan 22;16:1740174. doi: 10.3389/fpls.2025.1740174 (PMC12872850; doi:10.3389/fpls.2025.1740174)
Supplement: Supplementary file 2 [file Table1.docx]

Supplementary Material

**Supplementary Table 1.** Overview of each protocol's materials reagents and equipment.

| **Protocol** | **P1** | **P2** | **P3** |
| --- | --- | --- | --- |
| **Common materials** | Razor blade, stereoscopic and optic microscopes, dissecting needles*, tweezers*, pipettes, microscope slides, coverslips. | | |
| **Specific materials and equipment** | Ultrasonic cell disruptor or vortex mixer, Eppendorf tubes, cover slips 24 x 50 mm, micropipette, laboratory oven | Glass vials, plastic or glass jar, slide warming tray. | Glass vials, glass pipettes, Oven and aluminum trays (or carboard) for drying slides, small petri dishes, slide warming tray, laboratory oven |
| **Common reagents** | H₂O₂ (35 %), Acetic acid, distilled water | | |
| **Specific reagents** | Stains (safranin, Astra blue, toluidine blue, or Congo red), water as mounting medium and nail polish to seal. | Safranin, ethanol, a clearing agent that requires thorough dehydration, mounting resin. | Safranin and Astra blue, ethanol, and mounting resin low-water content compatible |

## *Dissecting needles can be made using sewing needles or fine insect pins with a cork or an eraser as handle. Sewing or craft tweezers work as well as the laboratory ones.

**Supplementary Table 2.** Comparative summary of protocols P1, P2, and P3, to assist in selecting, optimizing or combining protocols based on specific research needs.

| **Protocol** | **P1** | **P2** | **P3** |
| --- | --- | --- | --- |
| **Objective** | Rapid xylem or phloem maceration. | Effective xylem maceration in wide range of species and wood densities, with intense high contrast staining. | Xylem maceration with very rapid dehydration and staining, with few drops of ethanol and stains. |
| **Permanent slides** | no | yes | yes |
| **General steps** | | | |
| **Ethanol dehydration** | Not needed. | 70% - 100% ethanol.  6 changes, 24 hours each. | Drops of ethanol 50% - 100%. 4 changes, seconds each. |
| **Staining** | Homogenize the sample, mix the homogenized cells with a stain and dilute it with water.  Stains: safranin, Astra blue, Congo red or toluidine blue for xylem, and Astra blue or Congo red for phloem. | Safranin powder after dehydration.  Stain with a saturated solution of safranin in 100% ethanol. | Safranin and Astra blue (both liquids) before dehydration. Stain with few drops of safranin + Astra blue for 5 to 15 minutes or more if necessary. |
| **Mounting** | Water as mounting medium. | Mounting resin (requires thorough dehydration), dry at room temperature. | Mounting resin (suitable for use with up to 20% water content), oven (65°C). |
| **General timings** | | | |
| **Maceration** | 2-6 hours on oven (depends on species and tissue type). Cell separation (few seconds) before staining. | Minimum 24-48 hours with heating on slide warming tray. No maximum without heating (at room temperature), e.g. 1 month or more. | 24-48 hours boiling on a slide warming tray.  1 month or more without heating (at room temperature). |
| **Staining** | Staining for 5 min. | 24 hours or more if needed. | 1-15 minutes, or more if needed. |
| **Mounting** | A few minutes to mount sample in water. | 5-10 minutes to apply mounting resin, coverslip, and drying at room temperature. | A few minutes to mount sample with mounting resin. Drying in oven for observation: overnight if urgent, or 1 to 3 days; 3 months to full polymerization and safe long-term storage. |

**Supplementary Table 3**. Summary of advantages and disadvantages of each protocol.

| **Protocol** | **Advantages** | **Disadvantages** |
| --- | --- | --- |
| **P1** | Rapid maceration of both xylem and phloem, widely applicable for various gymnosperms and angiosperms species. Homogeneous distribution of cells on the slide that facilitates quantitative analysis. Does not require dehydration. A few minutes for staining and slide preparation. | Not suitable for permanent slides. Requires an ultrasonic homogenizer (or a vortex mixer) to reach optimal results. |
| **P2** | Tested in wide range of species. Suitable for both species with high and low wood densities, potentially effective for all taxons (gymnosperms and angiosperms). Produces excellent cell separation, intense staining and high-quality permanent slides for observation, imaging, and measuring. | Time-intensive process: extended maceration, dehydration and staining times (hours, days, weeks).  Requires thorough dehydration process to avoid cloudiness. |
| **P3** | Ideal for fast-paced research, very quick dehydration (seconds to few minutes) with few amounts of reagents (drops). Does not require clearing agent. Ideal for very small samples (reduces tissue loss during the process).  Produces high-quality permanent slides. | Requires the use of specific mounting resin to avoid cloudiness (tolerant to certain water content) due to non-extended dehydration. |
